# Chemical Oxidation of Coronene: A Crystallographic and Computational Study of Charge-Regulated π‑Stacking

**DOI:** 10.1021/acsphyschemau.6c00020

**Published:** 2026-03-18

**Authors:** Rameswar Bhattacharjee, Megan E. McCormack, Yikun Zhu, Zheng Wei, Miklos Kertesz, Marina A. Petrukhina

**Affiliations:** † Department of Chemistry and Institute of Soft Matter, 8368 Georgetown University, 37th and O Streets, NW, Washington, D.C. 20057-1227, United States; ‡ Department of Chemistry, University at Albany, State University of New York, 1400 Washington Avenue, Albany, New York 12222, United States

**Keywords:** Coronene, oxidation, π-stack, charge distribution, pancake bonding, DFT

## Abstract

Chemical oxidation of coronene with [Et_3_O^+^SbCl_6_^–^] in dichloromethane allowed the isolation and crystallization of a dimeric coronene radical cation in the form of [(C_24_H_12_)_2_^•+^(SbCl_6_^–^)]­[Et_3_O^+^SbCl_6_^–^]_2_, as revealed crystallographically. The crystal structure exhibits π-stacked columns of coronene molecules, wherein alternating average interplanar contacts of 3.357(2) and 3.452(2) Å show the distinct formation of the coronene dimers. This packing is accompanied by an atom-over-atom overlap motif and core deformation often associated with a unique intermolecular interaction called pancake bonding. A combined computational analysis employing finite cluster and periodic density functional theory (DFT) calculations confirms the presence of pancake bonding and delineates its hierarchical strength along the column. Charge-distribution analyses identify the molecular oxidation states and demonstrate that Coulombic repulsion between charged dimers plays a decisive role in enforcing the observed alternation of interplanar distances. Periodic DFT modeling of an infinite coronene stack faithfully reproduces the experimental structural parameters and provides insight into the electronic structure of the resulting one-dimensional π-radical assembly.

## Graphical abstract

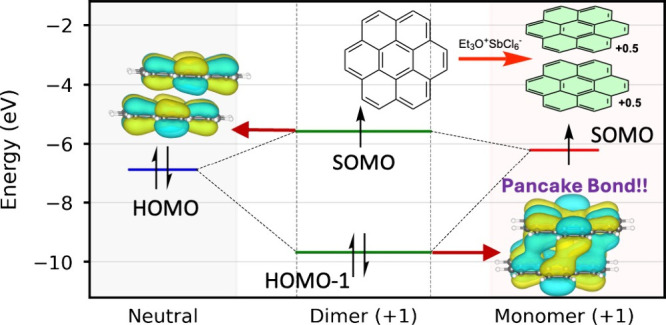

## Introduction

Since the isolation of graphene in 2004,^1^ polycyclic aromatic hydrocarbons (PAHs) have drawn great attention as models of the two-dimensional, π-conjugated surface.^2^ The unique properties of PAHs and easy modification of the polyarene cores allow potential applications in organic electronics, such as OLEDs and solar cells.^3,4^ Modification of intermolecular interactions through oxidation can enhance the properties of PAHs by introducing tunable conductivity and magnetism.^5^ Upon partial oxidation, the small PAHs generally exhibit a rearrangement from the herringbone-like packing motif of the neutral parents to conductive face-to-face π-stacks, opening electron transport pathways.^6,7^ One PAH, coronene (C_24_H_12_) with a highly symmetric (*D*_6*h*_) disk shaped core comprised of six aromatic six-membered rings, can be regarded as the smallest fragment of graphene.^8,9^ The great interest in coronene emerges from its unique properties, which are easily modulated through functionalization of the polyarene core^10–12^ or modification of the crystal packing through formation of charge-transfer complexes with various transition metals.^13–15^

Despite the great interest in coronene due to the properties^10–13^ and potential applications,^16–18^ its high chemical stability has hampered in-depth redox chemistry investigations. Specifically, the chemical oxidation of this readily available PAH is limited to *in situ* solution studies only.^19–22^ However, electrochemical oxidation of coronene by the Yoshida group afforded eight radical cation products with varying composition and charge distribution.^23–26^ In 2014, two products with the composition of [(C_24_H_12_)_3_^2+^(Mo_6_Cl_14_^2–^)] and [(C_24_H_12_)_3_^2+^(Mo_6_Br_14_^2–^)] were isolated and crystallographically characterized.^23^ In the solid-state, the coronene molecules are perpendicular to the metal clusters, with no face-to-face interactions between the hydrocarbon units. In 2016, four different products with the compositions of [(C_24_H_12_)_3_^2+^(Mo_6_O_19_^2–^)], [(C_24_H_12_)_3_^2+^(W_6_O_19_^2–^)],^25^ [(C_24_H_12_)^+^(GaCl_4_^–^)] and [(C_24_H_12_)_5_^2+^(GaCl_4_^–^)_2_]_,_ ^24^ were isolated and characterized. All these products were formed through electrochemical oxidation in the presence of the tetrabutylammonium salts of the respective counteranions. In 2019, electrochemical oxidation of coronene in the presence of [Et_4_N^+^GaBr_4_^–^] or [Et_4_N^+^FeBr_4_^–^] afforded two products with the compositions of [(C_24_H_12_)^+^(GaBr_4_^–^)] and [(C_24_H_12_)^+^(FeBr_4_^–^)].^26^ In the solid-state, the above six products exhibit continuous columns of coronene with interplanar distances below 3.4 Å, suggesting unusually strong π–π interactions. However, to the best of our knowledge, there has been no comprehensive investigation into the potential bonding interactions within these coronene π-stacks.

The presence of short C···C contacts below 3.4 Å (the van der Waals radius threshold) is characteristic of pancake bonding,^27,28^ an unconventional attractive interaction observed in oxidized PAHs and other planar π-radical species. Unlike conventional π-stacking, which is dominated by dispersion forces, pancake-bonded radical dimers engage in multicenter n-electron (ne/mc) interactions arising from significant SOMO–SOMO (singly occupied molecular orbitals) overlap, imparting partial covalent character to the intermolecular interaction.^29,30^ Previous studies have demonstrated that increasing the positive charge on π-radical dimers dramatically enhances their binding strength, with radical cation dimers exhibiting substantially stronger interactions than their neutral, closed-shell counterparts.^31–33^ However, the extent and strength of pancake bonding are sensitive to the oxidation state and electronic structure of the monomers.^34,35^ This unique coupling between charge and intermolecular bonding has been demonstrated in acene radical cation dimers and other π-radical systems. A defining feature of pancake-bonded PAH assemblies is their pronounced face-to-face, atom-over-atom alignment, reflecting the highly directional nature of this interaction.^27,36^

While the electrochemical oxidation of coronene has been developed rather extensively, the chemical oxidation remains underdeveloped due to the lack of reliable synthetic methods for this poorly soluble and highly stable PAH. To address this limitation, [Et_3_O^+^SbCl_6_^–^] was selected as a reliable strong oxidant^37,38^ for the controlled oxidation of coronene. This allowed the successful isolation of the first chemically oxidized product with the composition of [(C_24_H_12_)_2_^•+^(SbCl_6_^–^)] [Et_3_O^+^SbCl_6_^–^]_2_. The product was characterized through single crystal X-ray diffraction, EPR, ATR-IR and UV–vis spectroscopy, as well as conductivity measurements enabled by the apparent air stability of the crystalline product. The crystal structure reveals π-stacked columns of coronene molecules with alternating average interplanar separations of 3.357(2) and 3.452(2) Å, indicative of discrete dimer formation within the stacks. Whereas the longer separation lies within the expected van der Waals regime, the shorter contact falls below the C···C van der Waals threshold, signaling the presence of an unconventional intermolecular interaction. To elucidate the origin and implications of these short contacts, an integrated computational study combining finite-cluster and periodic density functional theory (DFT) calculations was performed. Coronene dimer, tetramer, and hexamer models elucidate the nature of bonding orbitals, spin delocalization, and energetic stabilization associated with discrete pancake-bonded units, while periodic calculations reveal how lattice constraints promote one-dimensional electronic delocalization in the extended columnar structure. This study integrates experimental and theoretical approaches to deliver a unified molecular-to-solid-state picture of how oxidation, stacking geometry, and counterion environment cooperatively govern the bonding and electronic landscape of coronene radical-cation materials.

## Results and Discussion

### Synthesis and Characterization of [(C_24_H_12_)_2_^•+^(SbCl_6_^–^)]­[Et_3_O^+^SbCl_6_^–^]_2_

One-pot mixing of coronene and [Et_3_O^+^SbCl_6_^–^] in a 1:1.5 ratio in anhydrous dichloromethane (DCM) at room temperature afforded a pale green suspension. Crystals suitable for X-ray diffraction analysis were grown upon slow diffusion of benzene into the DCM solution. After 7 days, thin green needles were deposited in ca. 50% yield. The X-ray diffraction analysis confirmed the product composition as [(C_24_H_12_)_2_^•+^(SbCl_6_^–^)] [Et_3_O^+^SbCl_6_^–^]_2_ (Figure 1). The crystals conform to a *P*–1 space group (Z = 1) with a unit cell volume of 1632.0(2) Å^3^.

**Figure 1:**
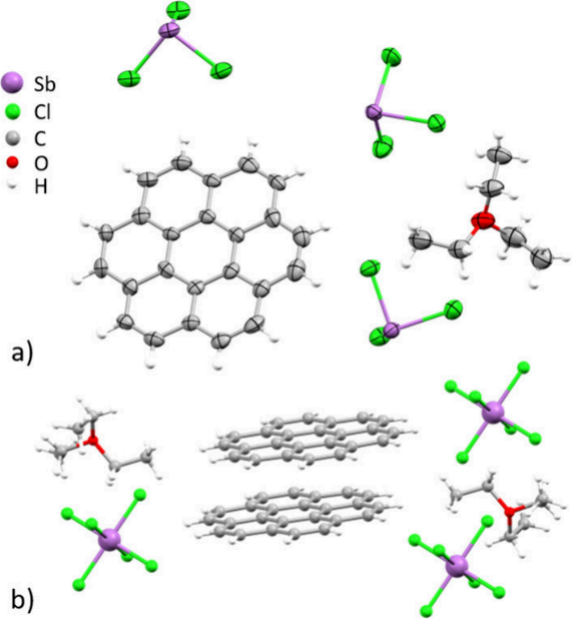
(a) Asymmetric unit of [(C_24_H_12_)_2_^•+^(SbCl_6_^–^)] [Et_3_O^+^SbCl_6_^–^]_2_, ORTEP drawing with thermal ellipsoids shown at the 50% probability level and (b) crystal structure of [(C_24_H_12_)_2_^•+^(SbCl_6_^–^)] [Et_3_O^+^SbCl_6_^–^]_2_, ball-and-stick model.

The formula unit shows two crystallographically equivalent coronene molecules per SbCl_6_^–^ anion, which cocrystallized with two equivalents of excess oxidant. The solid-state structure revealed continuous π-stacks of coronene units, which are isolated by surrounding SbCl_6_^–^ anions and cocrystallized oxidant (Figure 2a). Additionally, the neighboring coronene units exhibit good surface overlap (Figure 2b). Within the coronene stacks, alternating interplanar contacts propagate down the 1D column, illustrating the formation of distinct dimers (3.345(2)–3.384(2) Å) (Figure 2c). Notably, the cocrystallization of excess [Et_3_O^+^SbCl_6_^–^] was previously observed by the Kochi group during chemical oxidation of octamethylbiphenylene.^38^

**Figure 2:**
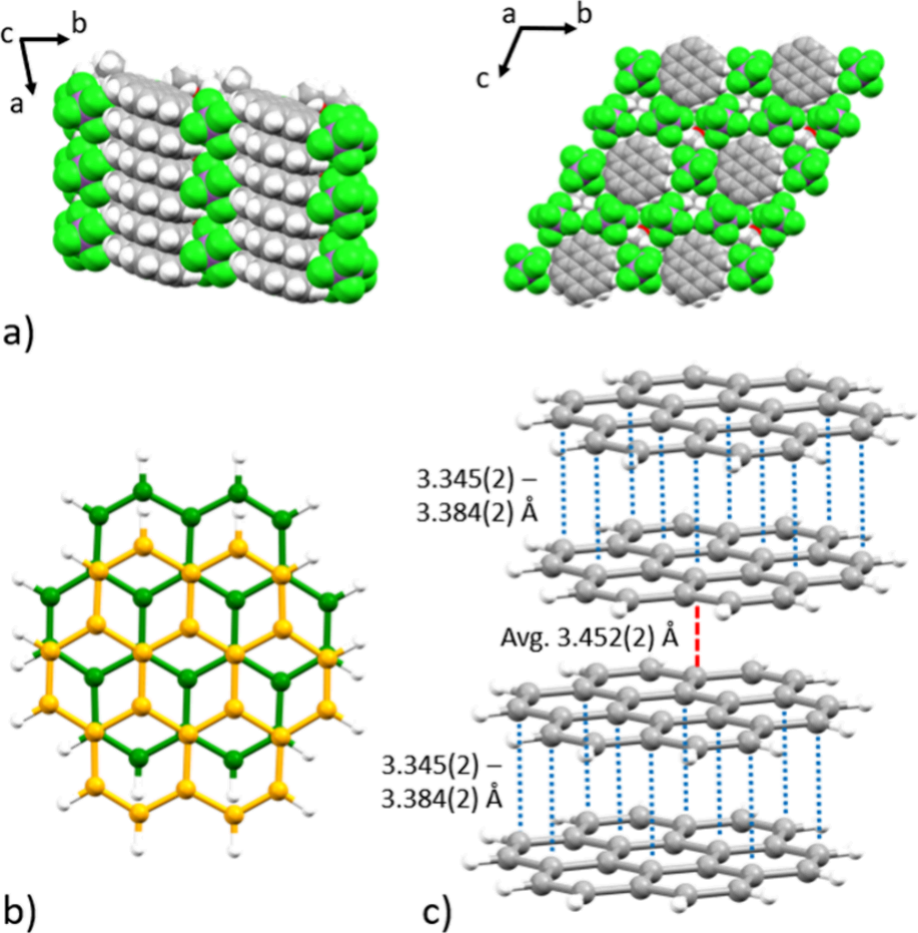
(a) Solid-state packing of [(C_24_H_12_)_2_^•+^(SbCl_6_^–^)] [Et_3_O^+^SbCl_6_^–^]_2_ down the *a*- and *c*-axis, (b) atom-over-atom overlap mode of neighboring molecules inside the coronene dimer units, and (c) interplanar contacts (Å) within π-stacks.

Each coronene unit exhibits three H···Cl interactions with two SbCl_6_^–^ anions, ranging from 2.877(2) to 2.948(2) Å (Figure 3a). Notably, one anion exhibits no intermolecular contacts with the coronene units and only interacts with the cocrystallized Et_3_O^+^ cations. Furthermore, the coronene molecules show clear core deformation upon oxidation, when compared to the reported crystal structure of the neutral parent,^39^ evident by the C–C bond length elongation, contraction, and changes in dihedral angles. The C–C bond elongation and contraction range from 0.015 to 0.035 Å and from 0.023 to 0.038 Å, respectively (Figure 3b), corresponding to the bonding/antibonding pattern of the highest occupied molecular orbital (HOMO) of neutral coronene. The dihedral angles between rings A/C and B/C are increased compared to the neutral parent from 0.80° to 1.25° and from 0.38° to 1.35°, respectively (Figure 3c), suggesting a slight decrease in planarity.

**Figure 3:**
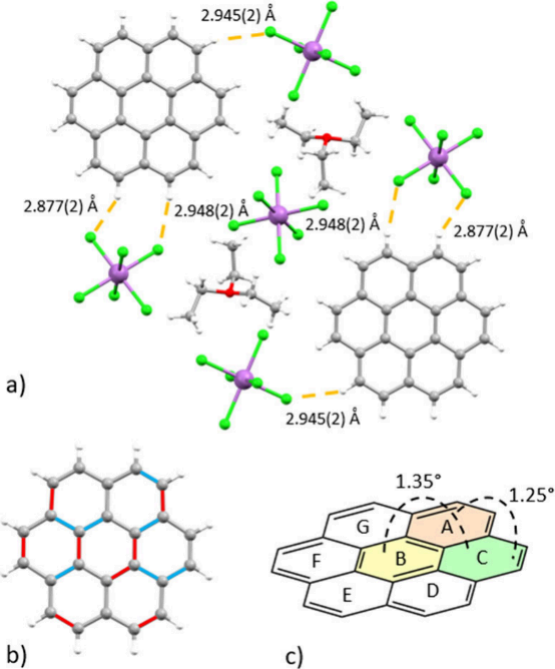
(a) H···Cl interactions, (b) sites of C–C bond elongation (highlighted in blue) and contraction (highlighted in red), and (c) dihedral angles within coronene units.

Additional product characterization was performed through EPR spectroscopy, UV–vis absorbance and diffuse reflectance spectroscopy, ATR-IR spectroscopy, and conductivity measurements. The EPR spectrum collected on crystals of [(C_24_H_12_)_2_^•+^(SbCl_6_^–^)]­[Et_3_O^+^SbCl_6_^–^]_2_ packed under argon exhibits an intense signal with a *g*-factor of 2.0034, characteristic of an organic radical (Figure 4). The UV–vis absorption measurements were carried out upon incremental addition of [Et_3_O^+^SbCl_6_^–^] to a solution of coronene, showing the gradual growth of a peak at 538 nm, which is assigned to the radical cation of coronene (Figure S1). Additionally, the solid-state diffuse reflectance spectra exhibit minimal changes over 24 h, suggesting the air stability of the product over this period (Figure S2). Due to this apparent air stability, conductivity was measured on the pressed pellet of the crystalline sample at room temperature, which yielded a value of 0.0072 S/cm. This value is lower than that of the five electrochemically oxidized coronene products prepared by the Yoshida group, which have the room temperature conductivities ranging from 0.4 S/cm to 3.0 S/cm.^24–26^ This can be attributed to the fact that these products show continuous columns with all interplanar contacts below 3.4 Å, while the title product exhibits distinct Peierls-like dimerization evident through crystallographic analysis. The ATR-IR spectra of both neutral coronene and the partially oxidized product are provided in the Supporting Information without further qualitative interpretation (Figure S3).

**Figure 4:**
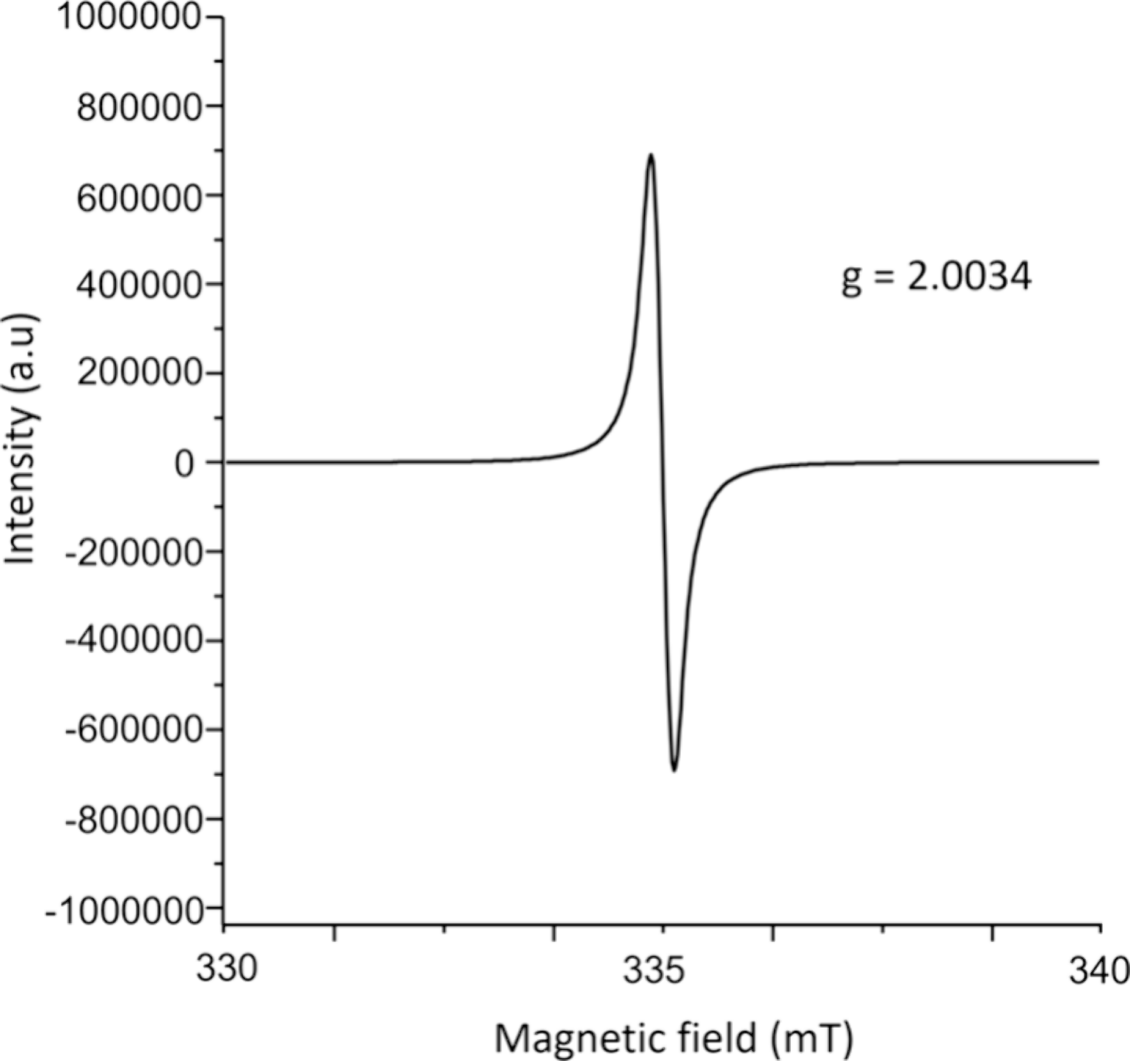
EPR spectrum of crystalline [(C_24_H_12_)_2_^•+^(SbCl_6_^–^)] [Et_3_O^+^SbCl_6_^–^]_2_, collected at 31.9 °C under Ar atmosphere.

### Computational Analysis

Previous studies have established that positively charged, open-shell PAHs tend to assemble into tightly packed π-stacks featuring shortened interplanar separations and enhanced intermolecular interaction energies.^33,40,41^ Such close packing is frequently attributed to significant intermolecular orbital overlap, often described as pancake bonding (PCB).^27,28,36^ Motivated by these observations, the intermolecular interactions within the present coronene radical-cation salt were examined in detail using DFT. Analysis of the crystal structure shows that coronene molecules assemble into continuous columnar stacks with alternating interplanar distances, such that each molecule interacts with two neighbors—one through a short contact and the other through a slightly longer separation. Consequently, the packing can be described as a sequence of coronene dimers, each carrying an overall +1 charge, as depicted in Figures 1 and 2.

To model this experimentally observed π-stacking motif, the dimer associated with the shorter intermolecular contact was isolated and optimized with a total charge of +1 (Figure 5a). The optimized geometry exhibits an average interplanar separation (d_av,_ eq 4) of 3.28 Å, only 0.07 Å shorter than the experimental value, representing excellent agreement given the absence of crystal-packing effects in the gas-phase calculation. Ten intermolecular C···C contacts shorter than 3.35 Å are identified within the optimized dimer, slightly below the conventional CC van der Waals limit of 3.4 Å. The spin-density distribution (Figure 5b) shows uniform delocalization of the unpaired electron across both coronene units, consistent with an even charge distribution that assigns an effective charge of +0.5 e to each monomer. This electronic symmetry is further supported by the presence of an inversion center between the two coronene molecules in the dimer, both in the XRD experimental structure and in the gas-phase optimized structure.

**Figure 5:**
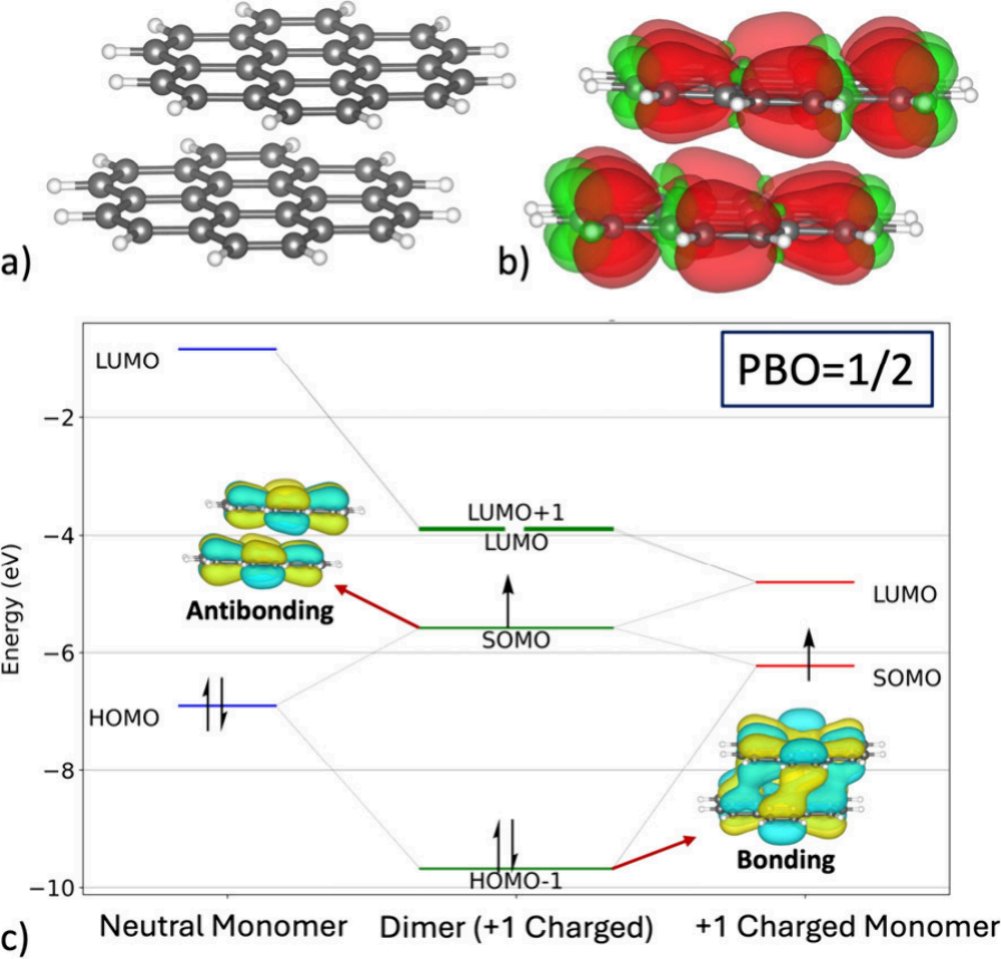
(a) Optimized geometry of the +1-charged coronene dimer [(C_24_H_12_)_2_]^+^. (b) Spin-density distribution (iso = 0.0005) for [(C_24_H_12_)_2_]^+^, highlighting the delocalization pattern across the stacked framework. (c) Simplified molecular-orbital diagram derived from the interaction of two valence orbitals from each monomer. Insets show the SOMO and HOMO–1 orbitals (iso = 0.015), whose formal occupancies are used to compute the pancake bond order (PBO).

Quantitative analysis of the intermolecular interaction energy further underscores the nondispersive nature of the bonding.^42^ The interaction energy for the +1-charged coronene dimer, Δ ***E***_*int*_^+1^(2) (eq 3), is calculated to be −28.1 kcal mol^–1^, substantially stronger than expected for a purely van der Waals interaction. For comparison, optimization of a neutral coronene dimer yields an interaction energy of Δ ***E***_*int*_^*o*^(2) = −16.3 kcal mol^–1^, in close agreement with the value of –17.6 kcal mol^–1^ reported by Janowski et al. using CCSD­(T) method.^42^ The resulting stabilization difference of 11.8 kcal mol^–1^ highlights the additional electronic contribution introduced upon oxidation, confirming that the interaction in the cationic dimer extends beyond dispersion and is consistent with significant pancake bonding character. In contrast, the +2-charged dimer is highly destabilized, with Δ ***E***_*int*_^+2^(2) = +29.7 kcal mol^–1^. Correspondingly, the intermonomer Coulomb repulsion (eq 2) increases from +20.9 to +53.1 kcal mol^–1^ upon going from +1 to +2 (Δ*V*_total_ = 32.2 kcal mol^–1^), demonstrating that electrostatic repulsion dominates over attractive interactions in the doubly charged species.^43^

Insight into the orbital origin of this stabilization is provided by a simplified molecular-orbital (MO) analysis constructed from the frontier valence orbitals of the two coronene monomers, yielding four corresponding dimer orbitals (Figure 5c). Interaction between the HOMO of the neutral monomer and the SOMO of the +1-charged monomer generates a bonding–antibonding pair. The bonding combination, corresponding to the HOMO–1 of the dimer, exhibits pronounced intermolecular orbital overlap characteristic of a pancake bond, while the antibonding counterpart becomes the SOMO of the dimer. Although the dimer SOMO is only weakly destabilized relative to the monomeric SOMO, the strong stabilization of the bonding HOMO–1 orbital dominates, resulting in a substantial net energetic gain. Because this interaction involves three electrons—two occupying the bonding orbital (HOMO–1) and one occupying the antibonding orbital (SOMO)—the formal pancake-bond order is 1/2, providing a physical explanation for the enhanced interaction energy observed in the +1-charged coronene dimer.

As evident from the crystal structure (Figure 2, *vide supra*), the coronene column displays a pronounced alternation in both interplanar distances (d_av_) and the number of short C···C contacts (m) along the stack. To elucidate the origin of this alternation, the computational analysis was extended to higher-order oligomers, specifically tetramers and hexamers. The total charge of each oligomer was assigned proportionally to the number of coronene dimer units (+2 for the tetramer and +3 for the hexamer). For the tetramer, the total interaction energy (Δ ***E***_*int*_^+2^(4)) is calculated to be −43.8 kcal mol^–1^. Although sizable, this value is significantly less stabilizing than predicted by simple dimer additivity. In an idealized additive model, a tetramer comprising two noninteracting dimers would be expected to exhibit an interaction energy of at least 2 × Δ ***E***_*int*_^+1^(2) = −56.2 kcal mol^–1^. The computed tetramer interaction energy is therefore ∼12.4 kcal mol^–1^ less stabilizing, indicating a clear interdimer penalty.

This reduction is well accounted for by electrostatic repulsion between the two positively charged dimer fragments (*V*_total_, eq 2 in ESI). The computed Coulombic repulsion between the +1/+1 dimer units is +12.9 kcal mol^–1^ (Figure 6a), in agreement with the 12.4 kcal mol^–1^ deviation from dimer additivity, noted above. The close correspondence between the Coulombic penalty and the observed energetic deficit indicates that neighboring dimers do not engage in additional stabilizing interactions beyond the intrinsic pancake bonding interactions within each dimer. The monomer-wise charge distribution (q, eq 1 in ESI) within the tetramer remains symmetric but nonuniform: the two terminal coronene units carry approximately +0.72 e each, whereas the two inner coronenes carry about +0.28 e each.

**Figure 6:**
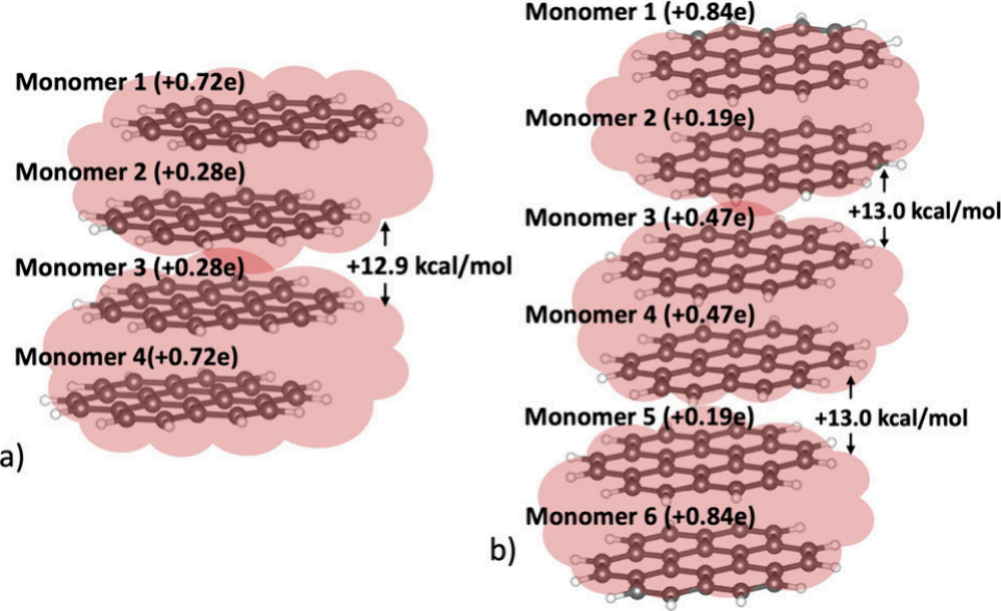
(a) Optimized geometry of the +2-charged coronene tetramer, [(C_24_H_12_)_4_]^2+^, showing monomer-wise charge distributions and the arrangement of two π-stacked coronene dimers. The total interdimer Coulombic repulsion (*V*_total_) is indicated. (b) Optimized geometry of the +3-charged coronene hexamer, [(C_24_H_12_)_6_]^3+^, with monomer-wise charge distributions, illustrating three interacting coronene dimers within the stack and the corresponding interdimer Coulombic repulsion.

A similar behavior is observed for the hexamer stack. The overall charge distribution again remains symmetric but nonuniform, with each dimer carrying approximately one unit of positive charge. In the central dimer, where both coronene units experience an equivalent local environment, the charge is evenly distributed (+0.47e per monomer). In contrast, the two terminal dimers exhibit the same polarization pattern observed in the tetramer, with the outermost coronene units bearing +0.84 e each and the adjacent inner units carrying only +0.19e. These results demonstrate that uniform charge distribution within a dimer arises only when both monomers reside in equivalent local environments, as in an isolated dimer, and the central dimer of the hexamer stack. Accordingly, an infinite coronene column is expected to approach a uniform charge distribution along its length. The symmetric but nonuniform charge distributions in the [(C_24_H_12_)_4_]^2+^ and [(C_24_H_12_)_6_]^3+^ clusters are fully consistent with the corresponding spin-density profiles shown in Figure S5 of the Supporting Information.

The total interaction energy of the hexamer, Δ ***E***_*int*_^+3^(6), is calculated to be –40.0 kcal mol^–1^, substantially smaller in magnitude than predicted by simple dimer additivity (3 × Δ ***E***_*int*_^+1^(2)) = –84.3 kcal mol^–1^). In the hexamer, two interdimer Coulombic repulsions are present, each contributing approximately +13.0 kcal mol^–1^ of destabilization, corresponding to an expected reduction of ∼26.0 kcal mol^–1^ in the total interaction energy. However, the actual loss of stabilization, quantified as Δ ***E***_*int*_^+3^(6) – 3 × Δ ***E***_*int*_^+1^(2) under the assumption of no additional interdimer interactions, is larger by 18.3 kcal mol^–1^. This additional energetic penalty is attributed to a weakening of the pancake-bond interactions in the two terminal dimers, arising from the highly uneven charge distributions in the terminal dimers.

Further evidence for the localized and extended nature of pancake bonding in coronene assemblies is provided by analysis of the key occupied molecular orbitals of the tetramer and hexamer (Figure 7a and d). Pronounced intermolecular orbital overlap is observed exclusively within individual dimeric units, whereas essentially no overlap appears between adjacent dimers, confirming that pancake bonds form only within the dimer pairs and are absent across dimer–dimer interfaces. This alternating bonding pattern is corroborated by structural metrics shown in Figure 7b, c, e, and f. Dimeric units engaged in pancake bonding exhibit a large number of short C···C contacts, whereas monomer pairs belonging to different dimers display only a few such contacts, consistent with weak van der Waals interactions. A parallel trend is observed in the average interplanar separations (d_av_), with the tetramer showing an interplanar distance alternation (IDA) of ∼0.070 Å and with the hexamer a slightly smaller value of ∼0.045 Å.

**Figure 7:**
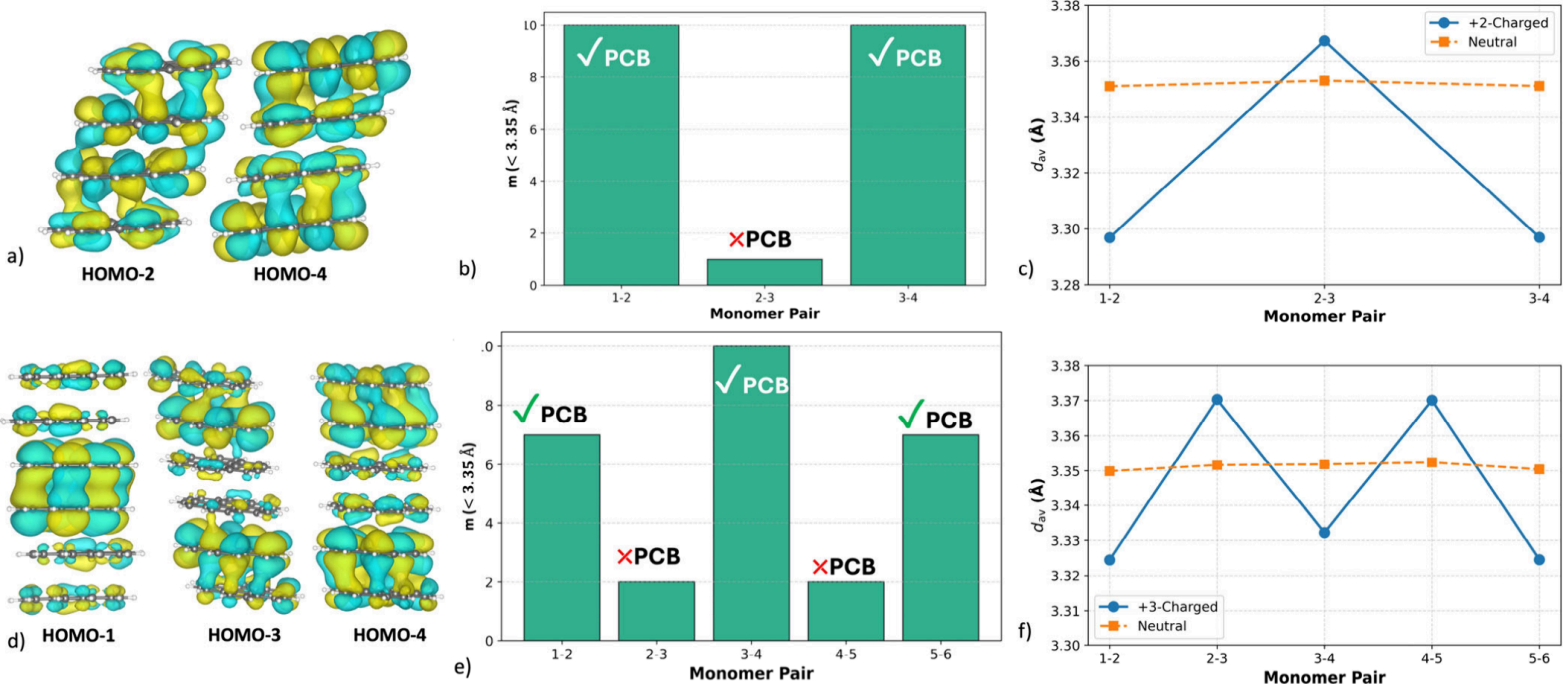
(a) Representative occupied molecular orbitals of the +2-charged coronene tetramer, highlighting the intermolecular π–π overlap within each stacked dimer pair. (b) Number of C···C short contacts, m (<3.35 Å) between coronene units in the tetramer. (c) Average optimized interlayer separation for each pair of adjacent coronenes in the +2-charged and neutral tetramer, as labeled in Figure 6a. (d) Representative occupied molecular orbitals of the +3-charged coronene hexamer showing the propagation of pancake-bonding interactions along the extended stack. (e) Number of C···C short contacts (m) in the hexamer structure. (f) Average optimized interlayer separation for each adjacent coronene pair in the +3- charged and neutral hexamer, as labeled in Figure 6b. All orbitals are plotted using iso value of 0.012.

Comparison of the charged clusters with their neutral analogues (Figure 7c and f), which serve as van der Waals reference systems, further supports this interpretation. For monomer pairs where pancake bonding is active, the d_av_ values in the charged systems fall below the corresponding neutral distances, indicating attractive stabilization. In contrast, at positions where pancake bonding is absent, the interplanar separations exceed the neutral reference values, reflecting repulsive interactions between adjacent dimer units. Key interaction energies, Δ ***E***_*int*_^*Q*^(*n*), and corresponding d_av_ values for both charged and neutral coronene stacks are summarized in Table 1. Taken together, these results demonstrate that extended coronene stacks adopt an alternating architecture composed of strongly interacting pancake-bonded dimers, separated by more weakly bound, van der Waals–dominated interfaces.

**Table 1:** Interaction Energies (*ΔE*_*int*_^*Q*^(*n*)), Interlayer Distances (*d*_*av*_), Short C···C Contact Counts (<3.35 Å), and Monomer Charge Distributions (q)^a^

n	Q	Δ*E*_*int*_^*Q*^(*n*) (kcal/mol)	*d*_*av*_ (Å)^a^	m < 3.35 Å	Charge distribution (q)
2	+1	–28.1	3.28	10	Uniform
2	0	–16.3	3.35	-	-
4	+2	–43.8	3.30	10	Symmetric, nonuniform
4	0	–47.9	3.35	-	-
6	+3	–40.0	3.32	(7, 10)^b^	Symmetric, nonuniform
6	0	–79.7	3.35	-	-

a Each entry corresponds to a system of the form [(*C*_24_ *H*_12_)_*n*_]^*Q*^, with varied number of coronenes in the stack (n), and total charge (Q).

b Short pair values are given; see Figure 7c and f.

c The hexamer contains two distinct types of short-contact pairs; therefore, two separate C···C contact counts are shown.

To elucidate how the local π–π interactions identified in finite clusters evolve upon infinite extension of the stack, periodic DFT calculations were performed to probe the resulting one-dimensional electronic structure. The periodic unit cell (Figure 8a) comprises two coronene molecules stacked along the crystallographic *a*-axis and carries a net charge of +1. The charge distribution is uniform across the dimer, with each coronene bearing approximately +0.5 charge, fully consistent with the gas-phase +1 dimer discussed earlier. The corresponding spin-density profile (Figure 8b) likewise reflects this even delocalization of the unpaired electron over both monomers.

**Figure 8:**
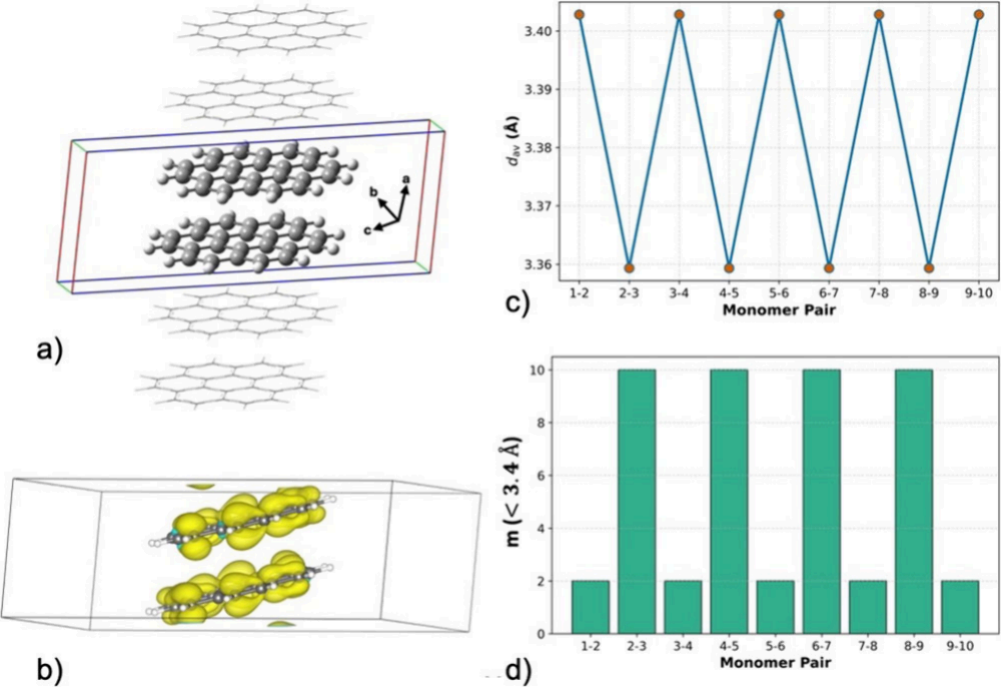
(a) Periodic +1-charged coronene dimer unit cell constructed from the experimental crystal structure after removal of counterions and excess oxidants. (b) Spin density plot (iso = 0.0008). (c) Alternation of the average interplanar separation along the infinite coronene column, showing a difference of approximately 0.06 Å between the short and long stacking pairs. (d) Number of C···C short contacts (<3.4 Å) for each stacking pair, highlighting the periodic alternation between strongly and weakly interacting coronene units.

The optimized periodic structure reproduces the key geometric signatures observed in the finite clusters: Figure 8b and d reveals a clear alternation in both d_av_ and the number of C···C short contacts. In the optimized geometry, the short pair exhibits an average separation of 3.35 Å and ten C···C contacts below 3.40 Å. In contrast, the long pair exhibits an average distance of 3.41 Å and only two such contacts. The resulting IDA of 0.06 Å in the periodic model mirrors that found in the discrete stacks. Periodic geometry also preserves the characteristic charge distribution of approximately +0.5 per coronene, reinforcing the finding of even charge distribution within the pancake bonded dimers in all oligomers discussed here (n = 2, 4, 6). Within this framework, the discrete HOMO–LUMO gaps predicted for finite clusters evolve into a shallow bandgap of 0.18 eV. This gap aligns remarkably well with the ∼0.13 eV semiconducting gap reported for analogous coronene-based materials.^24^

## Conclusion

We report the synthesis and solid-state characterization of a coronene radical-cation salt generated by chemical oxidation of coronene with [Et_3_O^+^SbCl_6_^–^] in dichloromethane. Single-crystal X-ray diffraction establishes the formation of a dimeric coronene radical cation, formulated as [(C_24_H_12_)_2_^•+^(SbCl_6_^–^)] [Et_3_O^+^SbCl_6_^–^]_2_, which assembles into extended π-stacked columns. Remarkably, the coronene layers do not adopt the uniform spacing typical of neutral π-stacks. Instead, the column exhibits a distinct alternation of short and long interlayer separations, revealing a structural modulation directly linked to electronic interactions within the charged stack. Consistent with the presence of an organic radical, the material shows an intense EPR signal (g = 2.0034).

Quantum-chemical analysis uncovers the origin of this unconventional stacking behavior. The computed interaction energy between adjacent coronene molecules greatly exceeds dispersion forces and is attributed to the formation of a three-electron “pancake bond”. However, this bonding is selectively expressed only in alternating pairs, producing a repeating dimer–dimer motif rather than a uniformly bonded π-chain. Molecular orbital analysis confirms a formal pancake-bond order of 1/2 within the strongly interacting pairs. The intervening pairs, which do not engage in pancake bonding, experience net electrostatic repulsion due to the positive charge distributed across all monomers. The interplay between strong pancake bonding, repulsive Coulomb interactions, and uniform monomer charge yields the experimentally observed alternating pattern of interlayer distances.

Periodic DFT calculations performed on an ion-stripped model of the crystal stack preserve this alternating geometry and reproduce the key features obtained from discrete cluster calculations. The periodic model also exhibits a homogeneous spin-density distribution across the two molecules within the unit cell, consistent with the gas-phase dimer radical. Although the full counterion environment is not included, the intrinsic coronene column displays a narrow electronic band gap of 0.18 eV, highlighting the profound electronic consequences of pancake bonding in charged polycyclic aromatics.

## Methods

### Experimental Section

All manipulations were carried out using break-and-seal^44^ and glovebox techniques under an atmosphere of argon. Dichloromethane (DCM, anhydrous, ≥ 99.8%) and benzene (Sigma-Aldrich) were dried over 4 Å molecular sieves and degassed. Coronene (95%) was purchased from Thermo Scientific and sublimed at 275 °C prior to use. Triethyloxonium hexachloroantimonate was purchased from Sigma-Aldrich and used as received. The UV–vis absorption spectra were recorded on a Shimadzu 2600i UV visible Spectrophotometer. The EPR spectrum was recorded on a LINEV ADANI Spinscan X Electron Paramagnetic Resonance Spectrometer. The UV–vis diffuse reflectance spectra were recorded on a Jasco V-770 Spectrophotometer. The IR spectrum was collected on a Shimadzu IRTracer-100 Fourier Transform Infrared Spectrometer QATR10 Single Reflection ATR accessory. Conductivity data were collected on a 2636B System Source Meter (Keithley Instruments Series) using a two-point probe method.

### Synthesis of [(C_24_H_12_)_2_^•+^(SbCl_6_^–^)]­[Et_3_O^+^SbCl_6_^–^]_2_

DCM (1.5 mL) was added to a custom-built glass system^45^ containing coronene (6.0 mg, 0.02 mmol) and [Et_3_O^+^SbCl_6_^–^] (13.1 mg, 0.03 mmol) under inert atmosphere. The mixture immediately turned to a pale olive green and was stirred at room temperature for 5 min. A small amount of dark green precipitate formed. The suspension was filtered to afford a golden solution. The golden solution was layered with anhydrous benzene (1.0 mL). The ampule was sealed under reduced pressure and left at 5 °C to produce dark green needles in 7 days. Yield: 9.5 mg, 54%. ATR-IR: 882, 1026, 1147, 1209, 1320, 1608, 3372 cm^–1^.

Single crystal X-ray diffraction data were collected on Rigaku XtaLAB Synergy-S X-ray diffractometer and processed by CrysAlisPro. The structure was solved by SHELXT and refined through the OLEX2 graphical interface (see ESI for more details).

### Computational

All gas-phase calculations were performed at the (U)­M05–2X^46^/6–311G­(d) level of theory. Unrestricted formalisms were employed for all open-shell systems. Geometry optimizations, electronic-structure calculations, and population analyses were carried out using Gaussian 16, Revision A.03.^47^ Molecular orbitals and spin-density distributions were visualized using the VESTA program.^48^

Atomic charges (*q*_*i*_) and molecular charge values (*q*) were obtained from Mulliken population analysis (eq 1).

1 $$q=\overset{l}{\underset{i}{\sum }}q_{i}$$

Here, *q*_*i*_ denotes the atomic charge on the atom *i*, and *q* is the total charge obtained by summing over all atomic charges of a molecule. The variable *l* represents the total number of atoms in a molecule.

Pairwise Coulombic repulsion energies were computed using a custom Python script in which all atom–atom electrostatic interactions between different coronenes were summed to yield the total repulsion energy *V*_total_ (eq 2).

2 $$V_{total}=\underset{i,j}{\sum }\frac{{332.06q}_{i}q_{j}}{r_{ij}}$$

where *i* and *j* refer to two atoms belonging to adjacent monomers. The electrostatic term uses the standard prefactor of 332.06 kcal·Å mol^–1^ e^–2^, converting Coulombic energy from atomic units into kcal/mol. In this expression, *q*_*i*_ and *q*_*j*_ are Mulliken charges (in e), *r*_*ij*_ is the interatomic separation (in Å). A cutoff distance of 15 Å was applied to ensure inclusion of all relevant, even weak, long-range interactions within each dimer.

Intercoronene interaction energies (**Δ** ***E***_*int*_^*n*^) within the stacks were evaluated using eq 3.

3 $${\Delta E}_{\mathrm{int}}^{Q}\left(n\right)=E_{[\left({C_{24}H_{12})}_{n}^{Q}\right]}-\frac{n}{2}\times E_{[\left({C_{24}H_{12})}_{1}^{2Q/n}\right]}-\frac{n}{2}\times E_{[\left({C_{24}H_{12})}_{1}^{0}\right]}$$

Here, *n* denotes the number of coronene units in the stack, and the total charge is *Q* = *n*/2 in the present system, as each dimer carries a + 1 charge. *E*_[(C24H12)_*n*_^*Q*^]_ denotes the total energy of the stack of *n* coronenes with a total charge of *Q*.

Interlayer distances (*d*_av_, eq 4) were evaluated using a custom Python script. A C···C cutoff distance of 3.35 Å, which is 0.05 Å shorter than its van der Waals limit, was used to isolate only those intermolecular contacts that fall below the vdW threshold and thus represent meaningful short contacts.

4 $$d_{av}=\overset{m}{\underset{i}{\sum }}\frac{d_{i}\left(\mathrm{C\cdot \cdot \cdot C}<\mathrm{cutoff}\right)}{m}$$

where *m* is the number of C···C short contacts between adjacent coronenes.

Periodic calculations were performed using PWSCF module (v7.1) of the Quantum Espresso package.^49^ All calculations employed spin-polarized DFT (n_spin_ = 2) with a total system charge of +1 and a target magnetization of 1.0 μ_B_. A unit cell derived from the experimental crystal structure—stripped of all counterions to isolate the coronene column—was employed to allow direct comparison with the isolated dimer, tetramer, and hexamer clusters. The lattice vector along the column axis was kept fixed at the experimental value, whereas the other two vectors were increased to 20 Å each to ensure no intercolumn interaction. The electronic wave functions were expanded using a plane-wave kinetic-energy cutoff of 70 Ry, and the charge density was expanded with a cutoff of 700 Ry. Brillouin-zone sampling was performed using a 10 × 1 × 1 Monkhorst–Pack k-point mesh.^50^ Ultrasoft pseudopotentials^51^ of PBE functional were used for the C and H atoms.^52^ All periodic structures were described using a fully general (ibrav = 0) cell. Dispersion interactions were treated using the Grimme’s D2 van der Waals correction.^53^ A convergence threshold of 1 × 10^–8^ Ry was applied for SCF iterations, with a mixing parameter of 0.3 and the Marzari–Vanderbilt cold smearing scheme with a degauss value of 0.02 Ry.^54^ Bader charge analysis was performed using the Bader program to quantify atomic charges within the periodic unit cell.^55^

## Supplementary Material





## Notes

X-ray diffraction, UV–vis, ATR-IR, EPR, and computational data (PDF)
Crystallographic information for [(C_24_H_12_)_2_^•+^(SbCl_6_^–^)] [Et_3_O^+^SbCl_6_^–^]_2_ (CIF)

## Notes

The deposition number 2518707 contains supplementary crystallographic data for this paper. These data can be obtained free of charge via the joint Cambridge Crystallographic Data Centre (CCDC) and Fachinformationszentrum Karlsruhe.
