# Supplementary material for: Chemical Oxidation of Coronene: A Crystallographic and Computational Study of Charge-Regulated π‑Stacking
Source: ACS Phys Chem Au. 2026 Mar 18;6(3):571–9. doi: 10.1021/acsphyschemau.6c00020 (PMC13220186; doi:10.1021/acsphyschemau.6c00020)
Supplement: Supplementary file 1 [file pg6c00020_si_001.pdf]

Supporting Information For

Chemical Oxidation of Coronene: A Crystallographic and Computational Study of Charge-Regulated π-Stacking

Rameswar Bhattacharjee,*[1] Megan E. McCormack,[2] Yikun Zhu,[2] Zheng Wei,[2] Miklos Kertesz,*[1] Marina A. Petrukhina*[2]

[1] Department of Chemistry and Institute of Soft Matter, Georgetown University, 37th and O Streets, NW, Washington, DC 20057-1227, USA.
E-mail: rb1820@georgetown.edu ,kertesz@georgetown.edu
[2] Department of Chemistry, University at Albany, State University of New York, 1400 Washington Avenue, Albany, NY 12222, USA.
E-mail: mpetrukhina@albany.edu

Table of Contents

I. Characterization of $[(C_{24}H_{12})_2^{\bullet+}(SbCl_6^-)][Et_3O^+SbCl_6^-]_2$ 3
I-A. EPR Spectroscopic Investigation 3
I-B. UV-Vis Spectroscopic Investigation 3
I-C. Conductivity Measurements 5
I-D. ATR-IR Spectroscopic Investigation 5
II. Crystal Structure Solution and Refinement 6
III. Spin Density Plots 11
IV. Optimized Coordinates: 12
V. References 33

I. Characterization of $[(C_{24}H_{12})_2^{\bullet+}(SbCl_6^-)][Et_3O^+SbCl_6^-]_2$

I-A. EPR Spectroscopic Investigation

Sample Preparation: Crystals of $[(C_{24}H_{12})_2^{\bullet+}(SbCl_6^-)][Et_3O^+SbCl_6^-]_2$ were transferred into the glovebox where solvent was removed. The dark green needles were dried *in-vacuo*. The crystals (2.0 mg) were loaded into a quartz capillary tube under argon. The tube was sealed, and the EPR spectrum was collected at 31.9 °C.

I-B. UV-Vis Spectroscopic Investigation

Sample Preparation: DCM (1.0 mL) was added to a glass ampule containing coronene (0.2 mg, 0.0006 mmol). Separately, DCM (1.0 mL) was added to a sealed flask containing $[Et_3O^+SbCl_6^-]$ (0.437 mg, 0.001 mmol). The UV-Vis absorption spectra were recorded at 25 °C during incremental addition of $[Et_3O^+SbCl_6^-]$ to a coronene solution in ratios of 1:1, 1:1.5, 1:2.5, 1:5, and 1:10.

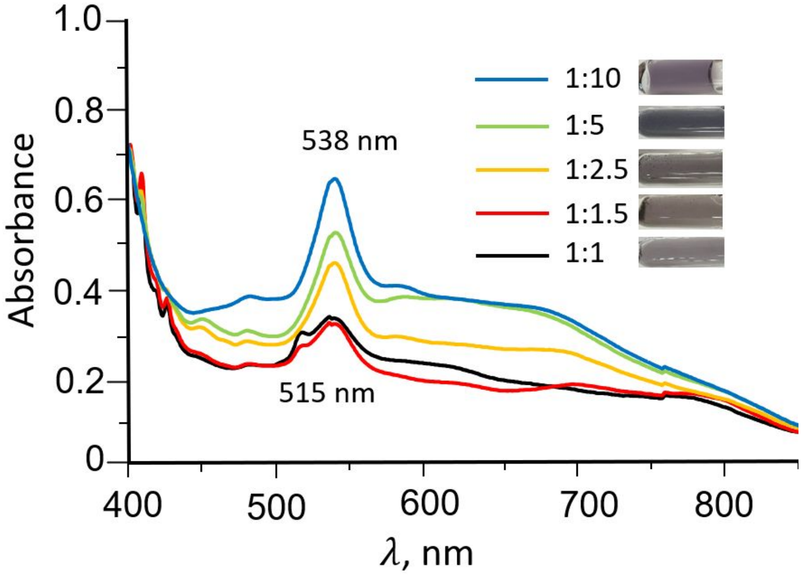

Figure S1. UV-Vis absorption spectra upon incremental addition of oxidant to a coronene solution in 1:1, 1:1.5, 1:2.5, 1:5, and 1:10 ratios in the 400 to 900 nm range.

Sample Preparation: Crystals of $[(C_{24}H_{12})_2^{\bullet+}(SbCl_6^-)][Et_3O^+SbCl_6^-]_2$ were transferred into the glovebox where solvent was removed. The dark green needles (5.0 mg) were dried *in-vacuo* and loaded into the sample holder under argon. The solid-state UV-Vis diffuse reflectance spectra were collected immediately after preparation and after 24 hours under air at 25 °C.

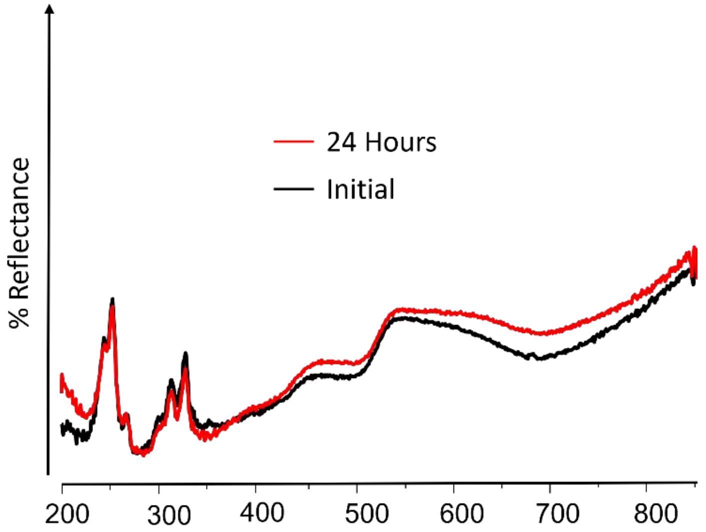

Figure S2. Diffuse reflectance spectra of $[(C_{24}H_{12})_2^{\bullet+}(SbCl_6^-)][Et_3O^+SbCl_6^-]_2$ immediately after removing from inert atmosphere and at 24 hours.

I-C. Conductivity Measurements

Sample Preparation: Crystals of $[(C_{24}H_{12})_2^{\bullet+}(SbCl_6^-)][Et_3O^+SbCl_6^-]_2$ were transferred to the glovebox where solvent was removed. The crystalline material was dried *in-vacuo*. The sample (54.6 mg) was loaded into a pellet press and compressed for a total of 10 minutes. The current-voltage curve was obtained using a two-point probe method at 25 °C.

I-D. ATR-IR Spectroscopic Investigation

Sample Preparation: Crystals of $[(C_{24}H_{12})_2^{\bullet+}(SbCl_6^-)][Et_3O^+SbCl_6^-]_2$ were transferred to the glovebox where solvent was removed. The crystals (4.0 mg) were dried *in-vacuo* and loaded onto the sample holder under argon. The sample holder was moved to the instrument, and the spectrum was collected at 25 °C. Separately, the ATR-IR spectrum of neutral coronene was collected under the same condition.

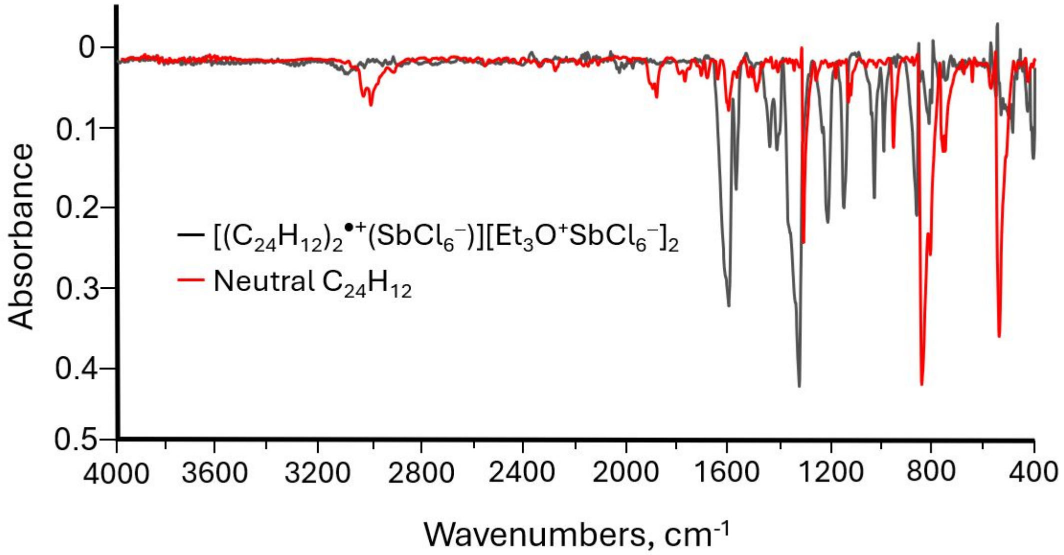

Figure S3. ATR-IR spectra of crystalline $[(C_{24}H_{12})_2^{\bullet+}(SbCl_6^-)][Et_3O^+SbCl_6^-]_2$ and neutral coronene.

II. Crystal Structure Solution and Refinement

Data collection of $[(C_{24}H_{12})_2^{\bullet+}(SbCl_6^-)][Et_3O^+SbCl_6^-]_2$ was performed at 100.15(10) K on a Rigaku XtaLAB Synergy-S X-ray diffractometer equipped with a HyPix-6000HE hybrid photon counting (HPC) detector and a microfocus Cu-Kα radiation (λ = 1.54184 Å). Data collection strategy to ensure completeness and desired redundancy were determined using CrysAlisPro.[1] Data processing was performed also using CrysAlisPro. Empirical absorption correction was applied using the SCALE3 ABSPACK scaling algorithm.[2] The structure was solved by SHELXT (version 2018/2)[3] and refined by full-matrix least-squares procedures using the Bruker SHELXTL (version 2019/3)[4] software package through the OLEX2 graphical interface.[5] All non-hydrogen atoms were refined anisotropically. Hydrogen atoms were included in idealized positions for structure factor calculations with U_{iso}(H) = 1.2 U_{eq}(C) and U_{iso}(H) = 1.5 U_{eq}(C) for methyl groups. In the structure of $[(C_{24}H_{12})_2^{\bullet+}(SbCl_6^-)][Et_3O^+SbCl_6^-]_2$, the triethyloxonium cation was found to be disordered. The disordered parts were modeled with two orientations with their relative occupancies refined. The geometries of the disordered parts were restrained to be similar. They were also restrained to have the same U_{ij} components, with a standard uncertainty of 0.01 Å^2. Further crystal and data collection details are listed in Table S1. The ORTEP drawing of the unit cell is shown in Fig. S4. The C–C bond distances and dihedral angles are shown in Tables S2 and S3, respectively.

Table S1. Crystallographic data of $[(C_{24}H_{12})_2^{\bullet+}(SbCl_6^-)][Et_3O^+SbCl_6^-]_2$.

Compound	$[(C_{24}H_{12})_2^{\bullet+}(SbCl_6^-)][Et_3O^+SbCl_6^-]_2$
Empirical formula	$C_{60}H_{54}Cl_{18}O_2Sb_3$
Formula weight	1810.38
Temperature (K)	100.15(10)
Wavelength (Å)	1.54184
Crystal system	Triclinic
Space group	*P*-1
a (Å)	7.1148(4)
b (Å)	16.1356(12)
c (Å)	16.4528(13)
α (°)	118.504(8)
β (°)	97.739(6)
γ (°)	92.526(5)
V (Å^3)	1632.0(2)
Z	1
ρ_{calcd} (g·cm^{-3})	1.842
μ (mm^{-1})	16.903
F(000)	889
Crystal size (mm)	0.04×0.04×0.36
θ range for data collection (°)	3.106–69.313
Reflections collected	14683
Independent reflections	5757 [R_{int} = 0.0825]
Transmission factors (min/max)	0.65208/1.00000
Data/restraints/params.	5757/156/419
$R1$,[a] $wR2$[b] ($I > 2\sigma(I)$)	0.0748, 0.1943
$R1$,[a] $wR2$[b] (all data)	0.1120, 0.2210
Quality-of-fit[c]	1.053

$R_{int} = \Sigma|F_o^2 - \langle F_o^2\rangle|/\Sigma|F_o^2|$

[a] $R1 = \Sigma||F_o|-|F_c||/\Sigma|F_o|$. [b] $wR2 = [\Sigma[w(F_o^2-F_c^2)^2]/\Sigma[w(F_o^2)^2]]$.

[c] Quality-of-fit = $[\Sigma[w(F_o^2-F_c^2)^2]/(N_{obs}-N_{params})]^{½}$, based on all data.

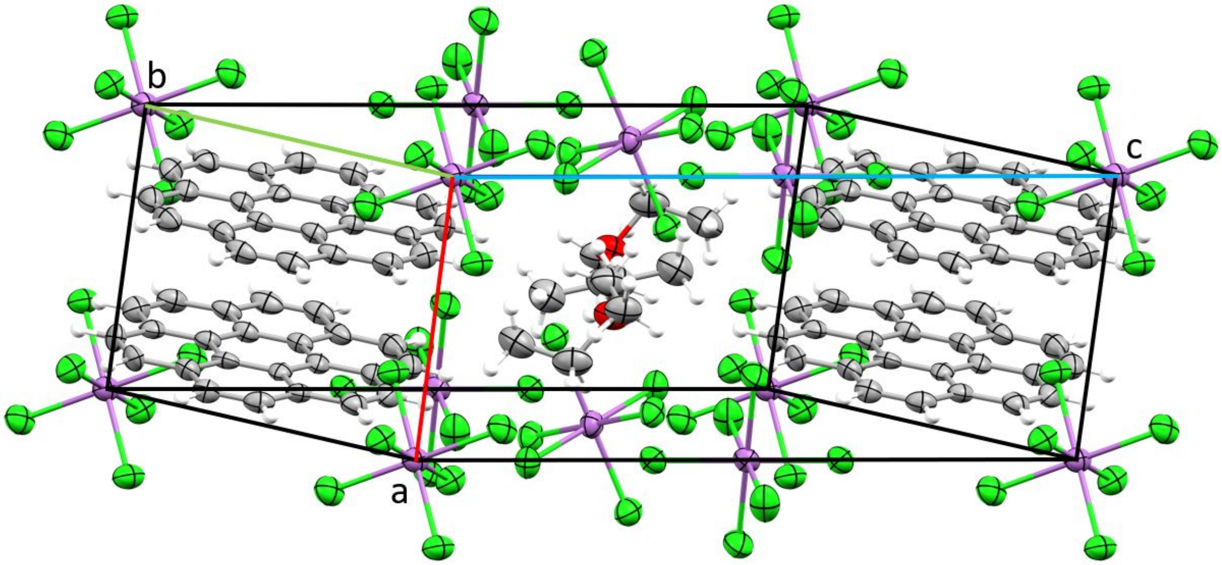

Figure S4. Unit cell of $[(C_{24}H_{12})_2^{\bullet+}(SbCl_6^-)][Et_3O^+SbCl_6^-]_2$, ORTEP drawing with thermal ellipsoids shown at the 50% probability level.

Table S2. C−C bond lengths (Å) of coronene in $[(C_{24}H_{12})_2^{\bullet+}(SbCl_6^-)]$ with a labeling scheme.

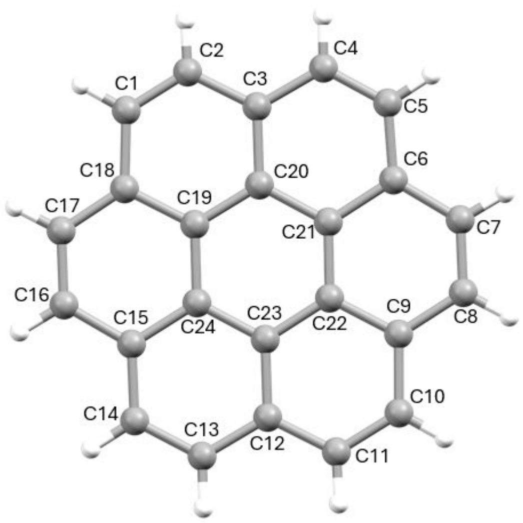

Bond	Distance (Å)	Bond	Distance (Å)
C1−C2	1.361(2)	C10−C11	1.338(2)
C2−C3	1.430(2)	C11−C12	1.425(2)
C3−C4	1.410(1)	C12−C13	1.415(2)
C3−C20	1.417(2)	C12−C23	1.421(2)
C4−C5	1.395(2)	C13−C14	1.340(2)
C5−C6	1.397(2)	C14−C15	1.409(2)
C6−C7	1.420(2)	C15−C16	1.421(2)
C6−C21	1.432(1)	C15−C24	1.438(1)
C21−C20	1.418(2)	C24−C23	1.432(2)
C20−C19	1.437(2)	C23−C22	1.396(1)
C19−C24	1.389(2)	C22−C21	1.401(2)
C19−C18	1.444(2)	C22−C9	1.428(2)
C18−C1	1.392(2)	C9−C10	1.407(2)
C18−C17	1.438(2)	C9−C8	1.447(1)
C17−C16	1.325(2)	C8−C7	1.350(2)

Table S3. Selected dihedral angles in neutral coronene and in $[(C_{24}H_{12})_2^{\bullet+}(SbCl_6^-)]$ with a labeling scheme.

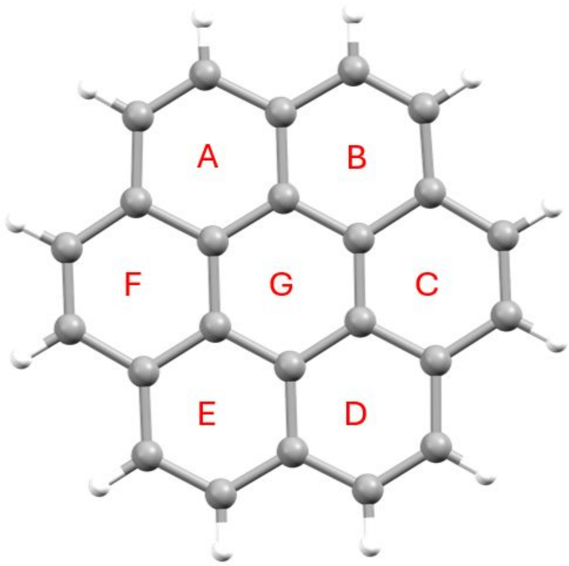

Ring	Cationic Coronene	Neat Coronene
**C/A**	0.45°	0.64°
**C/B**	1.25°	0.80°
**A/B**	0.19°	0.81°
**C/E**	0.99°	0.80°
**C/F**	0.84°	0°
**C/G**	1.35°	0.38°

III. Spin Density Plots

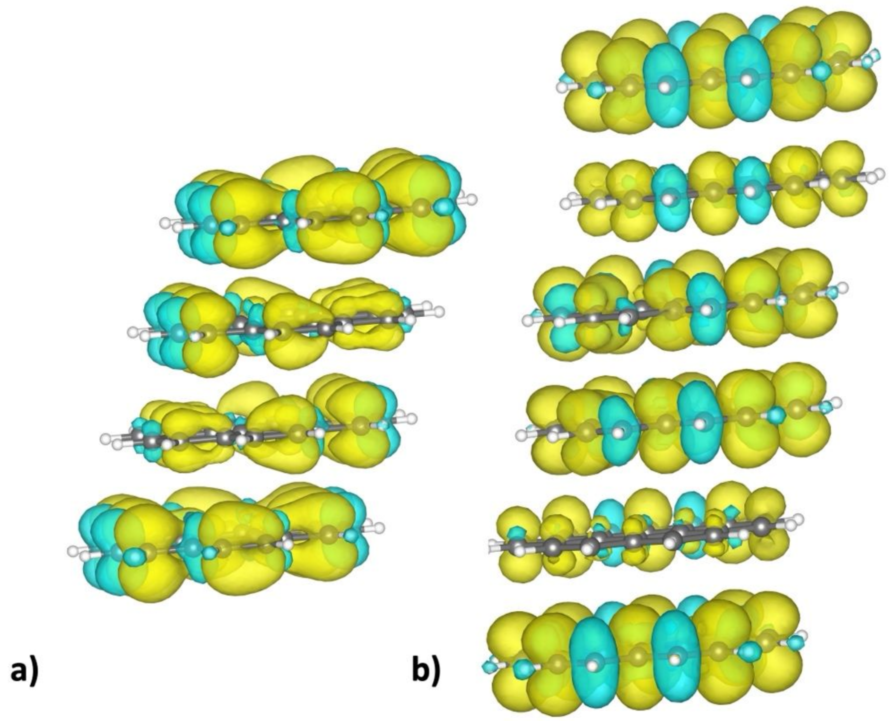

Figure S5. Spin density plot (iso=0.0005) of (a) $[(C_{24}H_{12})_4]^{2+}$, and (b) $[(C_{24}H_{12})_6]^{3+}$

IV. Optimized Coordinates:

$[(C_{24}H_{12})_2]^+$

72

C	2.41803400	0.00353400	0.93762700
C	1.74212500	1.22905400	1.15671900
C	-0.28798700	-0.00340800	1.78609200
C	0.38597100	1.22531000	1.57609800
C	-1.63817300	-0.00692300	2.21365500
C	1.74800800	-1.22538000	1.15513200
C	-0.28699800	2.44472300	1.79820800
C	0.39174500	-1.22859100	1.57431500
C	2.41717400	2.45310100	0.96570500
C	-2.28963900	-1.25029100	2.42844200
H	-3.31956500	-1.24783100	2.75910100
C	3.77856400	0.00706400	0.53135500
C	4.43859700	1.25118300	0.34485000
H	5.47808100	1.24947600	0.04471100
C	0.40812000	3.65461900	1.59608200
H	-0.10525300	4.59028300	1.77526300
C	3.79666400	-2.41459400	0.55175200
H	4.31861000	-3.35316500	0.41726300
C	-2.29550000	1.23309200	2.43031900
H	-3.32542900	1.22524400	2.76090000
C	-0.27549800	-2.45151300	1.79451600
C	-1.64784200	2.41376400	2.22420300
H	-2.15865000	3.35285100	2.39399700
C	4.44481500	-1.23372900	0.34388400
H	5.48437500	-1.22658000	0.04407600
C	1.72644400	3.65892700	1.19906900
H	2.24795900	4.59780800	1.06469500
C	3.78474300	2.42875000	0.55420400
H	4.30212700	3.36995200	0.42046800
C	-1.63642500	-2.42763600	2.22057700
H	-2.14286300	-3.36933800	2.38899900
C	2.42897900	-2.44597600	0.96261700
C	1.74388100	-3.65530900	1.19395500
H	2.26979700	-4.59156200	1.05838900
C	0.42537100	-3.65776300	1.59059200
H	-0.08363300	-4.59611500	1.76819500
C	-2.41805200	-0.00552800	-0.93781500
C	-1.74063900	-1.23002700	-1.15771500
C	0.28774600	0.00536000	-1.78683700
C	-0.38448100	-1.22428700	-1.57713900
C	1.63805200	0.01085200	-2.21391700
C	-1.74968300	1.22440100	-1.15482700

C	0.29027700	-2.44278500	-1.79921600
C	-0.39360300	1.22957100	-1.57444900
C	-2.41405900	-2.45502700	-0.96715800
C	2.28780400	1.25507500	-2.42834400
H	3.31779000	1.25415200	-2.75881200
C	-3.77842300	-0.01102900	-0.53099000
C	-4.43686600	-1.25597200	-0.34525000
H	-5.47627900	-1.25579300	-0.04486400
C	-0.40327200	-3.65362700	-1.59769100
H	0.11139700	-4.58855300	-1.77699900
C	-3.79956400	2.41067000	-0.54959600
H	-4.32257100	3.34851500	-0.41417300
C	2.29736000	-1.22830100	-2.43014300
H	3.32746700	-1.21890600	-2.76014300
C	0.27203600	2.45341600	-1.79400500
C	1.65133400	-2.40988000	-2.22453900
H	2.16357100	-3.34822200	-2.39412500
C	-4.44611200	1.22892300	-0.34224900
H	-5.48551900	1.22025000	-0.04194900
C	-1.72172600	-3.65983500	-1.20104200
H	-2.24200400	-4.59946900	-1.06712500
C	-3.78152500	-2.43265200	-0.55545300
H	-4.29772200	-3.37457200	-0.42217100
C	1.63291100	2.43150600	-2.22012500
H	2.13809200	3.37394900	-2.38818500
C	-2.43206000	2.44404200	-0.96116500
C	-1.74860700	3.65439000	-1.19204600
H	-2.27566900	4.58988100	-1.05568300
C	-0.43029100	3.65872800	-1.58916500
H	0.07752200	4.59780100	-1.76636800

$[(C_{24}H_{12})_2]$

72

C	2.66050700	-0.00273800	0.79074300
C	1.99909700	1.23049200	1.04486600
C	-0.00405000	0.00273100	1.78587300
C	0.66561100	1.23286900	1.53947800
C	-1.32651700	0.00543700	2.26858600
C	1.99473100	-1.23323700	1.04666600
C	0.00759900	2.45301200	1.78423700
C	0.66112700	-1.23012500	1.54094600
C	2.66234000	2.44837400	0.80340400
C	-1.97678100	-1.23610600	2.50051200
H	-2.99989200	-1.22826800	2.85356600
C	3.97554200	-0.00544000	0.28757500
C	4.62056400	1.23721800	0.04476100

H	5.63334100	1.23025400	-0.33889700
C	0.69940800	3.66789100	1.53399500
H	0.18631100	4.60310300	1.71980300
C	3.98082000	-2.43021200	0.30137200
H	4.48398500	-3.37182100	0.11956800
C	-1.97205400	1.24962600	2.49953700
H	-2.99512500	1.24592100	2.85277700
C	-0.00143500	-2.44756700	1.78690000
C	-1.32906600	2.42837500	2.26452300
H	-1.83691200	3.36989900	2.43251200
C	4.61626800	-1.25073500	0.04688300
H	5.62910000	-1.24791500	-0.33668200
C	1.97922700	3.66650200	1.06376200
H	2.49468500	4.60094500	0.87897900
C	3.98925500	2.41931700	0.29738600
H	4.49567200	3.35887100	0.11398600
C	-1.33818700	-2.41746700	2.26665000
H	-1.84969800	-3.35691300	2.43514700
C	2.65370000	-2.45382300	0.80715000
C	1.96616900	-3.66913000	1.06904300
H	2.47836700	-4.60567300	0.88583500
C	0.68610900	-3.66526700	1.53861600
H	0.16951900	-4.59835500	1.72540800
C	-2.66045200	0.00184200	-0.79062000
C	-1.99837300	-1.23095400	-1.04508500
C	0.00406800	-0.00184100	-1.78580700
C	-0.66484400	-1.23242900	-1.53956100
C	1.32656500	-0.00365900	-2.26843300
C	-1.99538100	1.23279000	-1.04624300
C	-0.00605800	-2.45212900	-1.78444300
C	-0.66187200	1.23057500	-1.54080000
C	-2.66101200	-2.44928600	-0.80416900
C	1.97591400	1.23831300	-2.50062500
H	2.99899600	1.23113300	-2.85377800
C	-3.97548900	0.00366700	-0.28745100
C	-4.61993300	-1.23941800	-0.04527100
H	-5.63275700	-1.23311300	0.33827300
C	-0.69726300	-3.66746400	-1.53476600
H	-0.18360400	-4.60233200	-1.72075100
C	-3.98202600	2.42844300	-0.30032700
H	-4.48566300	3.36971900	-0.11811400
C	1.97299600	-1.24742800	-2.49920800
H	2.99612700	-1.24305100	-2.85226300
C	-0.00017100	2.44846300	-1.78688400
C	1.33071300	-2.42660100	-2.26440900
H	1.83920300	-3.36778600	-2.43234900

C	-4.61682900	1.24853400	-0.04620400
H	-5.62962800	1.24504600	0.33744400
C	-1.97721500	-3.66694300	-1.06488700
H	-2.49222200	-4.60173500	-0.88060900
C	-3.98804100	-2.42109700	-0.29838700
H	-4.49404400	-3.36097600	-0.11550700
C	1.33646800	2.41926600	-2.26699000
H	1.84718100	3.35906000	-2.43595900
C	-2.65501500	2.45293700	-0.80632900
C	-1.96826800	3.66870700	-1.06815000
H	-2.48096300	4.60490500	-0.88457200
C	-0.68838600	3.66570500	-1.53820600
H	-0.17244400	4.59914000	-1.72505900

$[(C_{24}H_{12})_4]^{2+}$

144

C	1.23005500	-1.09967300	1.27169600
C	1.81415100	-1.84321100	0.21262800
C	2.42788200	0.25411800	-0.91876500
C	2.41097700	-1.16532100	-0.88416100
C	3.03315000	0.92750100	-2.00229400
C	1.23138100	0.31890800	1.23055000
C	2.98571100	-1.90227800	-1.93965400
C	1.83538900	0.99603600	0.13706400
C	1.80317400	-3.25221000	0.24870400
C	3.03531200	2.34787300	-2.01592000
H	3.50576100	2.85984300	-2.84551800
C	0.63074000	-1.77400300	2.36203800
C	0.63502500	-3.19497200	2.37814300
H	0.17338400	-3.70617700	3.21311100
C	2.95466100	-3.31701700	-1.88397000
H	3.39481400	-3.88150600	-2.69630600
C	0.03035300	0.35368500	3.35634900
H	-0.44329900	0.92347700	4.14542700
C	3.60945000	0.16354700	-3.05262400
H	4.07538500	0.68324500	-3.87985500
C	1.84514200	2.40496000	0.09966200
C	3.58725500	-1.19929600	-3.02241800
H	4.03361300	-1.76961400	-3.82748900
C	0.02847400	-1.00931700	3.39724600
H	-0.44366700	-1.52957500	4.22073500
C	2.37953900	-3.97093000	-0.82541600
H	2.36507200	-5.05338400	-0.79876800
C	1.20121200	-3.90738000	1.36237200
H	1.19677200	-4.98998200	1.38751200
C	2.46488400	3.05928800	-1.00204800

H	2.47192600	4.14189200	-1.02411800
C	0.63454300	1.05687900	2.27350100
C	0.64621800	2.46965500	2.20887600
H	0.17196800	3.03374200	3.00176000
C	1.23783600	3.12207900	1.15783000
H	1.23375700	4.20415900	1.11963400
C	5.83876100	0.83117700	-0.29976200
C	5.24808000	1.56501200	0.75456900
C	4.62001200	-0.53138700	1.86347300
C	4.63084500	0.88231200	1.83392800
C	4.01886300	-1.21503100	2.95486000
C	5.83136100	-0.58238400	-0.26976300
C	4.04619200	1.61408900	2.89064000
C	5.21746500	-1.26443500	0.81163400
C	5.28215900	2.97645600	0.74019200
C	4.03649800	-2.63376000	2.97332600
H	3.58478600	-3.14950700	3.80952500
C	6.47301400	1.51627500	-1.37508000
C	6.49517100	2.93555400	-1.37041100
H	6.98967500	3.45223800	-2.18192100
C	4.08815900	3.01716900	2.85121600
H	3.65035600	3.58010000	3.66485400
C	7.07351900	-0.60300200	-2.37969300
H	7.55580600	-1.16952100	-3.16549100
C	3.43405400	-0.45868300	4.00520900
H	2.98049600	-0.98381300	4.83458800
C	5.22144900	-2.67540100	0.84948900
C	3.43934500	0.90238700	3.97173200
H	2.99323100	1.46965200	4.77811200
C	7.08417500	0.75875100	-2.41100000
H	7.57572700	1.28301000	-3.21943200
C	4.69975700	3.68334500	1.80471300
H	4.73794000	4.76489100	1.80351200
C	5.92416500	3.64009800	-0.35196300
H	5.96138500	4.72162000	-0.35115300
C	4.60900000	-3.33827800	1.95717100
H	4.61939200	-4.42016700	1.98248800
C	6.44622700	-1.31557600	-1.30847600
C	6.43939900	-2.71827600	-1.24940500
H	6.92261800	-3.28237500	-2.03642100
C	5.83514500	-3.38265000	-0.19767100
H	5.84804500	-4.46413200	-0.16617000
C	-1.22757500	1.09201700	-1.27584000
C	-1.80991600	1.84198200	-0.22033800
C	-2.42851500	-0.24833400	0.92119100
C	-2.40896700	1.17081800	0.87936500

C	-3.03517800	-0.91513300	2.00800900
C	-1.23267000	-0.32629200	-1.22815000
C	-2.98265100	1.91419300	1.93093500
C	-1.83793600	-0.99658800	-0.13122200
C	-1.79595900	3.25077700	-0.26329100
C	-3.03966000	-2.33546600	2.02896800
H	-3.51066200	-2.84233200	2.86138200
C	-0.62627600	1.75973400	-2.36920200
C	-0.62726000	3.18060600	-2.39202000
H	-0.16412900	3.68678600	-3.22921200
C	-2.94877100	3.32856200	1.86824000
H	-3.38823500	3.89797600	2.67750700
C	-0.03266400	-0.37411200	-3.35435200
H	0.43842500	-0.94886300	-4.14137200
C	-3.61019700	-0.14487900	3.05433400
H	-4.07706200	-0.65949700	3.88420600
C	-1.85081000	-2.40532700	-0.08698600
C	-3.58565900	1.21780100	3.01710900
H	-4.03118200	1.79300300	3.81916500
C	-0.02651000	0.98866400	-3.40121100
H	0.44678800	1.50379500	-4.22725300
C	-2.37154300	3.97600600	0.80688800
H	-2.35481700	5.05828300	0.77487000
C	-1.19220900	3.89919100	-1.37990900
H	-1.18534500	4.98164200	-1.41032600
C	-2.47119700	-3.05300500	1.01836900
H	-2.48033600	-4.13547300	1.04573000
C	-0.63836200	-1.07065300	-2.26802300
C	-0.65355100	-2.48300200	-2.19669300
H	-0.18128300	-3.05207500	-2.98719900
C	-1.24602700	-3.12894200	-1.14207500
H	-1.24453300	-4.21082900	-1.09863800
C	-5.84020200	-0.82397700	0.30425600
C	-5.25136900	-1.56391100	-0.74686600
C	-4.61930600	0.52608700	-1.86560900
C	-4.63325200	-0.88747700	-1.82966000
C	-4.01700500	1.20342600	-2.96023600
C	-5.82997800	0.58944500	0.26768100
C	-4.05075500	-1.62530300	-2.88333400
C	-5.21499200	1.26525700	-0.81700800
C	-5.28839600	-2.97522200	-0.72600400
C	-4.03126400	2.62213800	-2.98500600
H	-3.57842900	3.13306100	-3.82355900
C	-6.47539000	-1.50279200	1.38297800
C	-6.50033300	-2.92207400	1.38494400
H	-6.99549200	-3.43396900	2.19908500

C	-4.09570100	-3.02811300	-2.83746400
H	-3.65961400	-3.59569800	-3.64879300
C	-7.07161800	0.62234400	2.37771100
H	-7.55263100	1.19345500	3.16096600
C	-3.43438600	0.44105000	-4.00740600
H	-2.97996300	0.96139000	-4.83932900
C	-5.21604100	2.67603500	-0.86137000
C	-3.44288500	-0.91986400	-3.96789600
H	-2.99853700	-1.49175000	-4.77198800
C	-7.08476000	-0.73924900	2.41549000
H	-7.57700800	-1.25875100	3.22656600
C	-4.70808100	-3.68821100	-1.78758000
H	-4.74856500	-4.76965500	-1.78143200
C	-5.93123900	-3.63248500	0.36955200
H	-5.97067400	-4.71392000	0.37375600
C	-4.60212600	3.33252200	-1.97204600
H	-4.61008600	4.41430600	-2.00223700
C	-6.44329700	1.32867800	1.30300200
C	-6.43389200	2.73110400	1.23728400
H	-6.91601200	3.29979000	2.02167500
C	-5.82839200	3.38937300	0.18248700
H	-5.83920300	4.47071700	0.14591200

$[(C_{24}H_{12})_4]$

144

C	1.10205500	-1.07188000	1.40366800
C	1.69624300	-1.90030100	0.41215500
C	2.45190700	0.10304400	-0.80671800
C	2.36706700	-1.31214700	-0.69543600
C	3.12859300	0.68504400	-1.89544400
C	1.17320600	0.34330500	1.28517000
C	2.94888900	-2.13121100	-1.68204600
C	1.85173700	0.93034800	0.18196100
C	1.61850000	-3.30113200	0.52568500
C	3.20324700	2.10081700	-1.98211000
H	3.74423200	2.54277600	-2.80944300
C	0.43982000	-1.65286400	2.50213100
C	0.38188700	-3.06880200	2.59731100
H	-0.13826700	-3.50974100	3.43883700
C	2.85037100	-3.54179500	-1.54794300
H	3.30801300	-4.16673200	-2.30515700
C	-0.10293300	0.55354100	3.34978300
H	-0.58752100	1.18823800	4.08084800
C	3.71622300	-0.16218000	-2.87221000
H	4.25439600	0.28994100	-3.69577900
C	1.92836400	2.33141600	0.06802300

C	3.62723700	-1.51910100	-2.77007800
H	4.09368100	-2.15411500	-3.51349800
C	-0.16329600	-0.80351800	3.46821100
H	-0.69527700	-1.25574800	4.29629300
C	2.21100500	-4.10606800	-0.48326500
H	2.14799100	-5.18381700	-0.39172100
C	0.94887000	-3.86360900	1.64473300
H	0.89164900	-4.94234100	1.72782800
C	2.62386100	2.89272900	-1.03536100
H	2.69131000	3.97116900	-1.10903200
C	0.56965900	1.16303400	2.25749400
C	0.65019800	2.57385600	2.11454000
H	0.16369300	3.19921900	2.85246900
C	1.30608800	3.13532900	1.05935100
H	1.35532800	4.21237800	0.95694300
C	6.07414000	0.74110600	-0.33513300
C	5.48373200	1.56678100	0.66109700
C	4.72736600	-0.43918300	1.87446300
C	4.80630100	0.97627300	1.76337100
C	4.04954700	-1.02314400	2.96135600
C	6.00227500	-0.67475700	-0.22026900
C	4.20987900	1.79271000	2.74266200
C	5.32754700	-1.26451500	0.88422000
C	5.56408000	2.96795500	0.55325600
C	3.97895900	-2.43924600	3.04744900
H	3.43858400	-2.88252600	3.87384000
C	6.72935000	1.32546500	-1.43596800
C	6.79133700	2.74229200	-1.52535800
H	7.30020900	3.18735000	-2.37159100
C	4.30604200	3.20349800	2.61124900
H	3.83944300	3.82577200	3.36447000
C	7.25060900	-0.87878600	-2.30170300
H	7.70512000	-1.51185400	-3.05378800
C	3.44592600	-0.17787400	3.93041100
H	2.90398600	-0.63191800	4.74990200
C	5.24988300	-2.66568600	0.99526100
C	3.52235600	1.17875700	3.82352200
H	3.04399900	1.81189300	4.56018500
C	7.31227500	0.47871000	-2.41690500
H	7.81818500	0.93275700	-3.26013100
C	4.96122500	3.77048900	1.55850700
H	5.02667100	4.84808400	1.47141300
C	6.23379300	3.53329000	-0.56455300
H	6.29394500	4.61183800	-0.64263700
C	4.55699500	-3.22991500	2.09947600
H	4.48293400	-4.30800900	2.16902700

C	6.59457100	-1.49218400	-1.20129400
C	6.50725900	-2.90356700	-1.06428800
H	6.96653000	-3.52821500	-1.82062200
C	5.85774800	-3.46802600	-0.00663200
H	5.78996600	-4.54508400	0.08196500
C	-1.10023000	1.06824200	-1.40614100
C	-1.69385500	1.89820800	-0.41559400
C	-2.45119500	-0.10322000	0.80536700
C	-2.36528200	1.31179000	0.69254000
C	-3.12851600	-0.68350100	1.89460700
C	-1.17228900	-0.34677000	-1.28599500
C	-2.94693800	2.13238600	1.67797600
C	-1.85135000	-0.93209000	-0.18220300
C	-1.61528300	3.29886000	-0.53079000
C	-3.20387600	-2.09913100	1.98307200
H	-3.74522800	-2.53974900	2.81088000
C	-0.43762300	1.64751300	-2.50528100
C	-0.37887200	3.06330200	-2.60216400
H	0.14155100	3.50293500	-3.44420600
C	-2.84747800	3.54275200	1.54226400
H	-3.30494500	4.16886100	2.29861100
C	0.10369500	-0.56024700	-3.35036300
H	0.58800500	-1.19611900	-4.08059100
C	-3.71586700	0.16523700	2.87022700
H	-4.25457000	-0.28557200	3.69417000
C	-1.92904800	-2.33297000	-0.06671200
C	-3.62598800	1.52198400	2.76653400
H	-4.09227500	2.15816100	3.50905300
C	0.16505300	0.79662800	-3.47029800
H	0.69753100	1.24753000	-4.29878500
C	-2.20747000	4.10536000	0.47710200
H	-2.14378300	5.18296100	0.38427300
C	-0.94535400	3.85959100	-1.65052500
H	-0.88749000	4.93818600	-1.73493100
C	-2.62489500	-2.89254200	1.03733000
H	-2.69297400	-3.97085500	1.11230100
C	-0.56941500	-1.16803600	-2.25743700
C	-0.65125200	-2.57863200	-2.11306700
H	-0.16537200	-3.20518900	-2.85039200
C	-1.30751700	-3.13844400	-1.05721800
H	-1.35768800	-4.21534400	-0.95367900
C	-6.07612000	-0.73645200	0.33807100
C	-5.48649400	-1.56457600	-0.65659200
C	-4.72784400	0.43835200	-1.87353100
C	-4.80828000	-0.97681000	-1.75985000
C	-4.04916100	1.01959700	-2.96134500

C	-6.00281000	0.67911700	0.22058000
C	-4.21254700	-1.79566900	-2.73754200
C	-5.32734100	1.26612900	-0.88491200
C	-5.56827900	-2.96546100	-0.54616600
C	-3.97707500	2.43546000	-3.05004200
H	-3.43602900	2.87664600	-3.87711500
C	-6.73192300	-1.31809400	1.43999100
C	-6.79530700	-2.73469300	1.53202700
H	-7.30462200	-3.17767400	2.37908400
C	-4.31024500	-3.20611000	-2.60357800
H	-3.84420200	-3.83025500	-3.35559200
C	-7.25092900	0.88829400	2.30163100
H	-7.70481500	1.52322200	3.05252400
C	-3.44620600	0.17191700	-3.92871200
H	-2.90356500	0.62388000	-4.74889100
C	-5.24815600	2.66700800	-0.99852200
C	-3.52414200	-1.18443300	-3.81938300
H	-3.04631300	-1.81943500	-4.55477700
C	-7.31398000	-0.46892300	2.41935500
H	-7.82035200	-0.92089000	3.26342000
C	-4.96615400	-3.77047200	-1.54987200
H	-5.03274000	-4.84783600	-1.46079600
C	-6.23858300	-3.52803800	0.57267800
H	-6.29981300	-4.60637700	0.65278100
C	-4.55447700	3.22847400	-2.10364700
H	-4.47926800	4.30636000	-2.17516900
C	-6.59426500	1.49897600	1.20008200
C	-6.50545200	2.91001400	1.06047700
H	-6.96411900	3.53654600	1.81561700
C	-5.85527300	3.47183300	0.00182600
H	-5.78635100	4.54865500	-0.08875100

$[(C_{24}H_{12})_6]^{3+}$

216

C	-2.22662900	-0.90885600	0.98001500
C	-1.71114000	-1.59079700	-0.15181000
C	-1.25703700	0.56224500	-1.24305500
C	-1.22539300	-0.85396900	-1.26342600
C	-0.76965600	1.29766000	-2.34934400
C	-2.26229100	0.50614400	0.99999800
C	-0.70930300	-1.53245800	-2.39011500
C	-1.77949300	1.24239600	-0.11324700
C	-1.68103000	-2.99900400	-0.17396500
C	-0.82641200	2.71192100	-2.31316300
H	-0.45491900	3.27153300	-3.16154500
C	-2.71907400	-1.64469800	2.08789800

C	-2.67457700	-3.05626600	2.04563500
H	-3.05552600	-3.61687800	2.88921900
C	-0.69603100	-2.94454700	-2.39161100
H	-0.30405400	-3.46380000	-3.25651000
C	-3.29061000	0.42088600	3.22386200
H	-3.71222300	0.94717900	4.07020700
C	-0.24700900	0.59216400	-3.47087700
H	0.13108300	1.15957600	-4.31122500
C	-1.82001500	2.65067900	-0.09666900
C	-0.21608000	-0.76813200	-3.49005000
H	0.18632500	-1.29324100	-4.34688100
C	-3.25961100	-0.93680100	3.20435600
H	-3.65479500	-1.50567000	4.03592500
C	-1.17030000	-3.65665600	-1.31776100
H	-1.15267900	-4.73923200	-1.33499600
C	-2.16848900	-3.71255000	0.95434400
H	-2.14380200	-4.79495500	0.93636700
C	-1.33455600	3.36590300	-1.22537900
H	-1.37311200	4.44786900	-1.20976200
C	-2.78726600	1.18591000	2.12409500
C	-2.81416000	2.59338500	2.12052200
H	-3.22761300	3.11389600	2.97436100
C	-2.34259000	3.30523200	1.04107400
H	-2.38237800	4.38716900	1.04895700
C	2.22804400	0.90645400	-0.98157900
C	1.71251000	1.58904000	0.14984900
C	1.25811200	-0.56336000	1.24219000
C	1.22666200	0.85286700	1.26184800
C	0.77027200	-1.29812500	2.34874700
C	2.26337400	-0.50854400	-1.00089800
C	0.71033900	1.53199300	2.38803800
C	1.78027800	-1.24415700	0.11266400
C	1.68220200	2.99726500	0.17109300
C	0.82604100	-2.71246200	2.31293700
H	0.45396600	-3.27159900	3.16137900
C	2.72050800	1.64166500	-2.08989100
C	2.67602700	3.05329100	-2.04843000
H	3.05705600	3.61339200	-2.89231600
C	0.69678600	2.94405300	2.38860500
H	0.30451200	3.46379900	3.25307000
C	3.29156700	-0.42455900	-3.22487600
H	3.71293500	-0.95134800	-4.07103300
C	0.24785500	-0.59202000	3.46998100
H	-0.13038000	-1.15896000	4.31058000
C	1.81978200	-2.65248600	0.09644900
C	0.21705900	0.76829000	3.48841400

H	-0.18534900	1.29391000	4.34493300
C	3.26095400	0.93315500	-3.20597300
H	3.65623400	1.50154100	-4.03782300
C	1.17108800	3.65555200	1.31432100
H	1.15311800	4.73813100	1.33078900
C	2.16975500	3.71017400	-0.95762500
H	2.14491100	4.79258200	-0.94029100
C	1.33364700	-3.36708600	1.22532400
H	1.37098900	-4.44910000	1.20978400
C	2.78793100	-1.18896000	-2.12479500
C	2.81394300	-2.59641200	-2.12077600
H	3.22696100	-3.11747000	-2.97449000
C	2.34182000	-3.30767000	-1.04111600
H	2.38079000	-4.38963900	-1.04878300
C	-4.94577500	1.23581100	-1.26557300
C	-5.45736000	1.86707900	-0.09990800
C	-5.89255900	-0.33949000	0.90257200
C	-5.92595400	1.07862500	0.98622600
C	-6.37920400	-1.12133900	1.97109800
C	-4.88966800	-0.18169600	-1.34097800
C	-6.42999100	1.70362800	2.14522400
C	-5.36889600	-0.96929100	-0.25874300
C	-5.50255800	3.27338100	-0.02116100
C	-6.33757200	-2.53718000	1.86277800
H	-6.72168400	-3.13380300	2.68038100
C	-4.48236400	2.01862800	-2.34674800
C	-4.54854500	3.43477400	-2.24810000
H	-4.19721000	4.03071300	-3.08083500
C	-6.45117800	3.12077400	2.20625400
H	-6.83605700	3.59870200	3.09837600
C	-3.89421500	0.00361700	-3.56123600
H	-3.48015600	-0.48009500	-4.43669900
C	-6.89002500	-0.46847400	3.12569200
H	-7.26865400	-1.07175800	3.94085200
C	-5.32874200	-2.37569100	-0.33926400
C	-6.91268500	0.89236000	3.21047000
H	-7.30750600	1.37671400	4.09489400
C	-3.95218500	1.36472600	-3.49179400
H	-3.58746900	1.96857700	-4.31305500
C	-6.00187400	3.87958100	1.15994900
H	-6.03049100	4.96045400	1.22065000
C	-5.04119600	4.03988600	-1.12864200
H	-5.08646700	5.12023000	-1.06824200
C	-5.83138800	-3.14089700	0.74901700
H	-5.80550000	-4.22132500	0.67930700
C	-4.36398700	-0.80745100	-2.48951500

C	-4.31940800	-2.22334800	-2.54121700
H	-3.90315400	-2.70065900	-3.41925200
C	-4.79088200	-2.98043500	-1.50389600
H	-4.75125800	-4.06129400	-1.55802000
C	-9.36972400	-1.03565600	0.55045400
C	-8.84888600	-1.65518400	-0.60757300
C	-8.38730800	0.54738000	-1.58063900
C	-8.34965000	-0.86192500	-1.67167200
C	-7.90939100	1.34380400	-2.65987400
C	-9.40890100	0.37329600	0.64190700
C	-7.83856900	-1.48048100	-2.83483600
C	-8.91422900	1.16605200	-0.42449200
C	-8.84084500	-3.06350200	-0.71367900
C	-7.97770300	2.75648200	-2.55704600
H	-7.62194200	3.35736800	-3.38243800
C	-9.88889700	-1.83285000	1.61263400
C	-9.86894400	-3.24604700	1.48691200
H	-10.27774600	-3.84729200	2.28765700
C	-7.83633100	-2.87974900	-2.91486300
H	-7.45697700	-3.35670300	-3.80873400
C	-10.46798800	0.16995000	2.84275000
H	-10.90035300	0.65007300	3.71071000
C	-7.39483500	0.70072000	-3.81779000
H	-7.03694500	1.31035000	-4.63589700
C	-8.97142100	2.57458100	-0.34249700
C	-7.35341500	-0.65709800	-3.90052600
H	-6.96457300	-1.13787100	-4.78850600
C	-10.43208900	-1.18873400	2.75784800
H	-10.83677700	-1.79710900	3.55528400
C	-8.33409900	-3.65537000	-1.87956100
H	-8.34201300	-4.73355800	-1.97165000
C	-9.36767800	-3.84058300	0.36623400
H	-9.37515100	-4.91864100	0.27331100
C	-8.48332900	3.35107800	-1.43941000
H	-8.53586700	4.42989500	-1.37288700
C	-9.95760800	0.99351200	1.78722700
C	-10.00416300	2.39283900	1.84717400
H	-10.43820400	2.87145900	2.71518100
C	-9.51665900	3.16713700	0.80580100
H	-9.57241800	4.24587300	0.86764000
C	4.94545000	-1.23567100	1.26606000
C	5.45692300	-1.86669500	0.10022700
C	5.89218400	0.34005100	-0.90179200
C	5.92554000	-1.07804500	-0.98574800
C	6.37883800	1.12212300	-1.97015600
C	4.88931100	0.18182300	1.34173400

C	6.42958500	-1.70280100	-2.14488300
C	5.36849700	0.96962200	0.25963400
C	5.50200900	-3.27298900	0.02114700
C	6.33714200	2.53793200	-1.86158500
H	6.72122000	3.13471900	-2.67908200
C	4.48212400	-2.01872300	2.34709900
C	4.54819900	-3.43485500	2.24811600
H	4.19686300	-4.03095000	3.08073800
C	6.45070700	-3.11993500	-2.20622200
H	6.83560100	-3.59770600	-3.09842400
C	3.89422800	-0.00393900	3.56209300
H	3.48035900	0.47959500	4.43774600
C	6.88973500	0.46948600	-3.12485900
H	7.26843400	1.07292500	-3.93986900
C	5.32829200	2.37600700	0.34041300
C	6.91237500	-0.89131900	-3.20991800
H	7.30723600	-1.37549900	-4.09442200
C	3.95220400	-1.36505100	3.49238000
H	3.58765000	-1.96905100	4.31360400
C	6.00132900	-3.87893900	-1.16009600
H	6.02988500	-4.95980200	-1.22103400
C	5.04069800	-4.03973900	1.12847800
H	5.08587900	-5.12007200	1.06781800
C	5.83093200	3.14142600	-0.74771300
H	5.80499100	4.22183900	-0.67782200
C	4.36375600	0.80734200	2.49044800
C	4.31902100	2.22324500	2.54235700
H	3.90275400	2.70037500	3.42048500
C	4.79036800	2.98053100	1.50514200
H	4.75064400	4.06137800	1.55941300
C	9.36941800	1.03721000	-0.54950900
C	8.84860300	1.65659000	0.60860000
C	8.38700700	-0.54611800	1.58137000
C	8.34940900	0.86319200	1.67260700
C	7.90907100	-1.34270000	2.66050400
C	9.40856500	-0.37172900	-0.64115000
C	7.83842800	1.48157000	2.83588200
C	8.91386100	-1.16463000	0.42513000
C	8.84053700	3.06491300	0.71487200
C	7.97725100	-2.75541100	2.55742500
H	7.62142900	-3.35638500	3.38272700
C	9.88851700	1.83455200	-1.61162600
C	9.86854400	3.24779000	-1.48572600
H	10.27731900	3.84910900	-2.28642900
C	7.83616700	2.88083100	2.91607500
H	7.45685500	3.35766900	3.81002900

C	10.46757100	-0.16809400	-2.84196100
H	10.89991700	-0.64808800	-3.71000300
C	7.39469400	-0.69979000	3.81853800
H	7.03682300	-1.30949600	4.63659600
C	8.97095700	-2.57317000	0.34289800
C	7.35335900	0.65805000	3.90147900
H	6.96462200	1.13868800	4.78958200
C	10.43165400	1.19062200	-2.75690200
H	10.83631600	1.79907500	-3.55429000
C	8.33384100	3.65661200	1.88082200
H	8.34172900	4.73478700	1.97305800
C	9.36734200	3.84216300	-0.36499600
H	9.37480500	4.92020600	-0.27191100
C	8.48278200	-3.34984000	1.43970800
H	8.53523300	-4.42864900	1.37298700
C	9.95722500	-0.99177600	-1.78656000
C	10.00371800	-2.39110500	-1.84671200
H	10.43772500	-2.86961100	-2.71480100
C	9.51616300	-3.16555900	-0.80546500
H	9.57187100	-4.24428600	-0.86748200

$[(C_{24}H_{12})_6]$

216

C	-2.38350100	-0.99507800	1.06383300
C	-1.85729900	-1.76476300	-0.00997500
C	-1.26141400	0.30757100	-1.20155900
C	-1.29921600	-1.11272000	-1.14389600
C	-0.69583600	0.95265400	-2.31765600
C	-2.35729900	0.42535900	1.00153400
C	-0.78243700	-1.87354500	-2.21013600
C	-1.79278500	1.07622800	-0.12969700
C	-1.88838400	-3.17074500	0.04966200
C	-0.66657400	2.37229600	-2.35029800
H	-0.20881200	2.86264200	-3.20023000
C	-2.93351000	-1.63944000	2.18857100
C	-2.94793200	-3.05909700	2.22674100
H	-3.38335200	-3.54865700	3.08929300
C	-0.83092800	-3.29064100	-2.12798700
H	-0.42011100	-3.87033100	-2.94556700
C	-3.45085000	0.51406400	3.17677400
H	-3.88328400	1.10421300	3.97490700
C	-0.17060800	0.16354900	-3.37524000
H	0.28774700	0.66412300	-4.21877900
C	-1.76047800	2.48235400	-0.18881000
C	-0.21425400	-1.19836000	-3.32356200
H	0.20774600	-1.78868000	-4.12776900

C	-3.46961600	-0.84792700	3.23970100
H	-3.91706700	-1.34869400	4.08962400
C	-1.36277800	-3.91624700	-1.03889300
H	-1.38788900	-4.99826200	-0.98786600
C	-2.44503000	-3.79748000	1.19602900
H	-2.46782700	-4.87998600	1.23667000
C	-1.18029700	3.10795100	-1.32376800
H	-1.14546700	4.18998800	-1.35572600
C	-2.89085200	1.18699600	2.05839800
C	-2.85456200	2.60416700	1.97195000
H	-3.28628700	3.18472400	2.77754900
C	-2.30877000	3.22682500	0.88869300
H	-2.29221800	4.30821700	0.83049700
C	2.38627400	1.02575300	-1.05951300
C	1.86779600	1.81146000	0.00648200
C	1.26024900	-0.24279300	1.22322100
C	1.30730300	1.17638800	1.14890900
C	0.69153200	-0.87105100	2.34733900
C	2.35100100	-0.39358200	-0.98040000
C	0.79675700	1.95298400	2.20671600
C	1.78490800	-1.02742100	0.15966700
C	1.90820400	3.21635000	-0.06963100
C	0.65179500	-2.29005700	2.39603600
H	0.19091600	-2.76747700	3.25163200
C	2.93701400	1.65316500	-2.19343400
C	2.96108300	3.07216000	-2.24811500
H	3.39704800	3.54867800	-3.11768100
C	0.85464400	3.36869100	2.10802900
H	0.44922900	3.96059200	2.91954300
C	3.43648900	-0.51508200	-3.15810500
H	3.86241100	-1.11743200	-3.95058700
C	0.17238500	-0.06620100	3.39596100
H	-0.28885500	-0.55391100	4.24544900
C	1.74383000	-2.43251900	0.23548900
C	0.22494300	1.29472000	3.32830100
H	-0.19223500	1.89728500	4.12589500
C	3.46406000	0.84589600	-3.23704000
H	3.91162400	1.33380600	-4.09436200
C	1.38924200	3.97797000	1.01096000
H	1.42179700	5.05911000	0.94735500
C	2.46645800	3.82591000	-1.22444700
H	2.49633300	4.90768300	-1.27782200
C	1.16004200	-3.04097800	1.37799300
H	1.11764200	-4.12228600	1.42257400
C	2.87650200	-1.17108100	-2.02974900
C	2.83303900	-2.58684800	-1.92567300

H	3.25947000	-3.17969600	-2.72511500
C	2.28633300	-3.19309300	-0.83370500
H	2.26377000	-4.27358800	-0.76248000
C	-4.88340700	1.17372500	-1.42613000
C	-5.41635700	1.92773600	-0.34443400
C	-6.00122300	-0.16201500	0.82217400
C	-5.97153000	1.25913500	0.78139100
C	-6.56418500	-0.82369900	1.92997200
C	-4.89976400	-0.24727600	-1.37972200
C	-6.49330200	2.00442700	1.85626100
C	-5.46256700	-0.91480100	-0.25741400
C	-5.39333700	3.33444600	-0.38767200
C	-6.58667800	-2.24384400	1.94486500
H	-7.04096800	-2.74727600	2.78907000
C	-4.33572900	1.83432600	-2.54244600
C	-4.33001700	3.25441700	-2.56429500
H	-3.89604100	3.75642400	-3.42031400
C	-6.45208400	3.42268100	1.79111000
H	-6.86360400	3.99073200	2.61657800
C	-3.79987600	-0.30410400	-3.55289900
H	-3.35950000	-0.88217100	-4.35539800
C	-7.09312300	-0.04999400	2.99716400
H	-7.54492700	-0.56339800	3.83660800
C	-5.48528700	-2.32171200	-0.21443500
C	-7.05679800	1.31270100	2.96189900
H	-7.47802300	1.89122100	3.77519200
C	-3.79154200	1.05852700	-3.60099000
H	-3.34545500	1.57197600	-4.44392400
C	-5.92371200	4.06402000	0.70931500
H	-5.90281400	5.14665900	0.67193300
C	-4.83878400	3.97785800	-1.52586900
H	-4.82195500	5.06082200	-1.55378000
C	-6.06578300	-2.96410700	0.91101300
H	-6.09216400	-4.04667000	0.93111600
C	-4.35704000	-0.99319500	-2.44313300
C	-4.38086400	-2.41146300	-2.37143100
H	-3.93969700	-2.97981200	-3.18051100
C	-4.92582700	-3.05013000	-1.29727200
H	-4.93217000	-4.13213900	-1.25040500
C	-9.62687100	-0.92666500	0.61321400
C	-9.09513700	-1.67270700	-0.47473500
C	-8.51195600	0.42488400	-1.62723800
C	-8.53338500	-0.99648400	-1.59263300
C	-7.94873000	1.09367700	-2.73049800
C	-9.61251500	0.49509500	0.57560400
C	-7.99519700	-1.73396300	-2.66406500

C	-9.05338400	1.17055500	-0.54422700
C	-9.11892800	-3.07976000	-0.44260000
C	-7.93339700	2.51398700	-2.73941500
H	-7.47976400	3.02255000	-3.58022100
C	-10.16750000	-1.59583000	1.72789300
C	-10.17441600	-3.01675800	1.73907100
H	-10.59540800	-3.52704800	2.59671500
C	-8.03187800	-3.15248500	-2.60739700
H	-7.60929800	-3.71375300	-3.43122900
C	-10.68740800	0.53555300	2.76083900
H	-11.09770500	1.10818000	3.58350900
C	-7.40211700	0.32717100	-3.79429800
H	-6.94725100	0.84629400	-4.62795400
C	-9.03270000	2.57765300	-0.57947600
C	-7.42330000	-1.03518900	-3.76064300
H	-6.98828500	-1.60778200	-4.57013800
C	-10.69374600	-0.82760500	2.80124400
H	-11.11155200	-1.34665700	3.65518100
C	-8.57610800	-3.80166400	-1.53907000
H	-8.59713900	-4.88417100	-1.50979500
C	-9.67320600	-3.73136400	0.69134500
H	-9.69031700	-4.81411500	0.71091400
C	-8.45510200	3.22822900	-1.70233600
H	-8.42331100	4.31055300	-1.71468100
C	-10.14719500	1.23368400	1.64807200
C	-10.11905600	2.65278900	1.58697000
H	-10.53337000	3.21660900	2.41379300
C	-9.58024100	3.29939900	0.51428600
H	-9.55601400	4.38151600	0.48360300
C	4.87057300	-1.12152700	1.46541300
C	5.38477700	-1.91700000	0.40467200
C	5.99918700	0.12598600	-0.82772400
C	5.94502300	-1.29257200	-0.74375600
C	6.56655600	0.74395200	-1.95829100
C	4.91133500	0.29699800	1.37596000
C	6.44668600	-2.07904200	-1.79865400
C	5.47994500	0.92033600	0.23140200
C	5.33764000	-3.32119200	0.49061700
C	6.61263300	2.16245400	-2.01676300
H	7.06989900	2.63213900	-2.87863500
C	4.31686800	-1.73847200	2.60357500
C	4.28737800	-3.15690600	2.66870900
H	3.84900900	-3.62528400	3.54136100
C	6.38129700	-3.49378000	-1.69032800
H	6.77774100	-4.09353300	-2.50060200
C	3.82230600	0.43854900	3.55078500

H	3.39599500	1.04811900	4.33741400
C	7.07480600	-0.07070400	-3.00472200
H	7.52978800	0.40905700	-3.86215900
C	5.52692400	2.32469200	0.14575400
C	7.01472100	-1.43094900	-2.92813500
H	7.42036400	-2.04086300	-3.72626900
C	3.79051500	-0.92163500	3.64014200
H	3.33909500	-1.40141200	4.49990000
C	5.84875300	-4.09278700	-0.58667700
H	5.80942700	-5.17326800	-0.51623500
C	4.77857000	-3.92015600	1.65055500
H	4.74326100	-5.00131600	1.71149300
C	6.11103100	2.92263000	-1.00215300
H	6.15582600	4.00347100	-1.05520000
C	4.38708500	1.08407100	2.41873300
C	4.43661000	2.49883100	2.30474800
H	4.01037000	3.09895200	3.09874900
C	4.98717800	3.09517600	1.20923500
H	5.01305700	4.17498900	1.12982600
C	9.64150500	0.81409500	-0.67158200
C	9.13448300	1.61360700	0.38983200
C	8.51293400	-0.42395900	1.62687700
C	8.56587300	0.99413400	1.53683700
C	7.94227500	-1.03655300	2.75863600
C	9.59615200	-0.60444900	-0.57829900
C	8.05222700	1.78462200	2.58219100
C	9.03009200	-1.22312100	0.57048300
C	9.18921900	3.01744100	0.30262500
C	7.89492000	-2.45482000	2.82273800
H	7.43530000	-2.92007400	3.68510700
C	10.18842700	1.42707600	-1.81515700
C	10.22624600	2.84597400	-1.88202700
H	10.65187200	3.31297200	-2.76175400
C	8.12034800	3.19872300	2.47016400
H	7.71697800	3.80089400	3.27451100
C	10.65404300	-0.75394200	-2.76712200
H	11.04589900	-1.36707200	-3.56933300
C	7.42053200	-0.21723300	3.79511000
H	6.95961600	-0.69323000	4.65090100
C	8.97844500	-2.62696100	0.66075100
C	7.47249900	1.14204600	3.70859800
H	7.05657500	1.75527100	4.49810700
C	10.69001200	0.60610100	-2.86088600
H	11.11303200	1.08201000	-3.73709400
C	8.67083000	3.79345700	1.37375400
H	8.71595400	4.87324600	1.30214500

C	9.74898900	3.61186500	-0.85965100
H	9.78975900	4.69237100	-0.92173000
C	8.39374700	-3.22031600	1.81138300
H	8.33768300	-4.30033300	1.86599600
C	10.10652300	-1.39600600	-1.62451000
C	10.04776700	-2.81061400	-1.50768700
H	10.44366800	-3.41524600	-2.31444700
C	9.50244600	-3.40276400	-0.40723900
H	9.45435000	-4.48203400	-0.33417200

Optimized Periodic Unit Cell containing Two Coronene with a total +1 charge, $[(C_{24}H_{12})_2]^+$

72

C	0.4501165096	3.6962105234	8.6306272200
C	0.3567622725	4.5607721185	7.5067714791
C	-0.0889841807	5.8997984973	7.6764141066
C	-0.9677570322	8.5624335403	8.0037026340
H	-1.3108250584	9.5887604362	8.1375580730
C	0.8894438026	2.3495690409	8.4591636555
C	1.1523763896	2.7413850214	6.0687320143
H	1.4264459480	2.3853468818	5.0751184938
C	-0.1943946460	6.7626501849	6.5496396606
C	1.2389655020	1.9031330926	7.1547557759
H	1.5839092810	0.8764576302	7.0304387807
C	-0.6355765924	8.0975385058	6.7492122937
H	-0.7101401847	8.7579391062	5.8845163431
C	0.7067839483	4.0853813075	6.2099236503
C	0.5891709926	4.9632000404	5.1035780649
H	0.8559460921	4.5950725140	4.1123881715
C	0.1449018959	6.2579178219	5.2678955534
H	0.0530911688	6.9195924048	4.4061105088
C	3.4346223125	6.1354181259	7.3725924157
C	3.9732746731	3.9309735867	8.3268144193
C	3.8756460367	4.7945364818	7.2033885847
C	4.4230173809	2.5886235585	8.1569902233
C	4.2296923265	4.3185307892	5.9065655613
C	3.3403984891	7.0021760112	6.2468414967
C	2.5786903231	8.8065254034	7.7013999444
H	2.2517052078	9.8385406818	7.8360808724
C	4.1214930950	5.2006548712	4.8011682349
H	4.3921659755	4.8349815292	3.8098576349
C	4.7765936265	2.1415798072	6.8531504411
H	5.1342357682	1.1191682785	6.7298554047
C	4.6870455657	2.9789384796	5.7664721879
H	4.9724201654	2.6272551902	4.7744342464
C	3.6832725997	6.4985651698	4.9644068707

H	3.5996857679	7.1604956367	4.1007541621
C	2.9119502024	8.3429163924	6.4476029489
H	2.8490109156	9.0059439144	5.5834047190
C	0.1055090475	4.1726502941	9.9244992443
C	-0.4331433131	6.3770948333	8.9702772407
C	-0.3355146767	5.5135319382	10.0937030753
C	-0.8828860309	7.7194448615	9.1401014467
C	-0.6895609665	5.9895376408	11.3905260987
C	0.1997328709	3.3058924188	11.0502501633
C	0.9614410369	1.5015430266	9.5956917156
H	1.2884261422	0.4695277382	9.4610107876
C	-0.5813617350	5.1074135488	12.4959234351
H	-0.8520346255	5.4730869008	13.4872340251
C	-1.2364622765	8.1664886128	10.4439412189
H	-1.5941044082	9.1889001415	10.5672362553
C	-1.1469142157	7.3291299404	11.5306194721
H	-1.4322888154	7.6808132298	12.5226574136
C	-0.1431412397	3.8095032502	12.3326847993
H	-0.0595544179	3.1475727833	13.1963374979
C	0.6281811576	1.9651520376	10.8494887111
H	0.6911204344	1.3021245156	11.7136869410
C	3.0900148504	6.6118578966	8.6664644400
C	3.1833690775	5.7472963015	9.7903201809
C	3.6291155407	4.4082699227	9.6206775534
C	4.5078883922	1.7456348797	9.2933890260
H	4.8509564184	0.7193079838	9.1595335970
C	2.6506875574	7.9584993891	8.8379280045
C	2.3877549604	7.5666833986	11.2283596457
H	2.1136854020	7.9227215382	12.2219731662
C	3.7345259960	3.5454182351	10.7474519994
C	2.3011658480	8.4049353274	10.1423358841
H	1.9562220690	9.4316107898	10.2666528793
C	4.1757079524	2.2105299142	10.5478793763
H	4.2502715347	1.5501293238	11.4125753169
C	2.8333474117	6.2226871125	11.0871680197
C	2.9509603674	5.3448683796	12.1935136051
H	2.6841852679	5.7129959060	13.1847034985
C	3.3952294641	4.0501505981	12.0291961066
H	3.4870401912	3.3884760152	12.8909811512

Lattice parameters (in Å)

7.1148000000	0.0000000000	0.0000000
-0.88145474	19.98056650	0.00000000
-2.69321391	-9.67249808	17.29709166

V. References

(1) Rigaku Corporation. Rigaku Oxford Diffraction, CrysAlisPro Software System, Version 1.171.43.134a. . **2024**.

(2) Rigaku Oxford Diffraction. SCALE3 ABSPACK; A Rigaku Oxford Diffraction Program (1.0.11,Gui:1.0.7) (C). . **2005**.

(3) Sheldrick, G. M. SHELXT–Integrated space-group and crystal-structure determination. *Acta Crystallogr. Sect. Found. Adv.* **2015**, *71* (1), 3-8.

(4) Sheldrick, G. M. Crystal structure refinement with SHELXL. *Acta Crystallogr. Sect. C Struct. Chem* **2015**, *71* (1), 3-8.

(5) Dolomanov, O. V.; Bourhis, L. J.; Gildea, R. J.; Howard, J. A.; Puschmann, H. OLEX2: a complete structure solution, refinement and analysis program. *J. Appl. Crystallogr* **2009**, *42* (2), 339-341.
